# Systematic discovery of gene fusions in pediatric cancer by integrating RNA-seq and WGS

**DOI:** 10.1186/s12885-023-11054-3

**Published:** 2023-07-03

**Authors:** Ianthe A. E. M. van Belzen, Casey Cai, Marc van Tuil, Shashi Badloe, Eric Strengman, Alex Janse, Eugène T. P. Verwiel, Douwe F. M. van der Leest, Lennart Kester, Jan J. Molenaar, Jules Meijerink, Jarno Drost, Weng Chuan Peng, Hindrik H. D. Kerstens, Bastiaan B. J. Tops, Frank C. P. Holstege, Patrick Kemmeren, Jayne Y. Hehir-Kwa

**Affiliations:** 1 Princess Máxima Center for Pediatric Oncology, Utrecht, The Netherlands; 2 Department of Pharmaceutical Sciences, Utrecht University, Utrecht, The Netherlands; 3 Oncode Institute, Utrecht, The Netherlands; 4 Center for Molecular Medicine, UMC Utrecht and Utrecht University, Utrecht, The Netherlands

**Keywords:** Gene fusions, Chimeric transcripts, Pediatric cancer, Structural variants, Whole genome sequencing, RNA sequencing

## Abstract

**Background:**

Gene fusions are important cancer drivers in pediatric cancer and their accurate detection is essential for diagnosis and treatment. Clinical decision-making requires high confidence and precision of detection. Recent developments show RNA sequencing (RNA-seq) is promising for genome-wide detection of fusion products but hindered by many false positives that require extensive manual curation and impede discovery of pathogenic fusions.

**Methods:**

We developed Fusion-sq to overcome existing disadvantages of detecting gene fusions. Fusion-sq integrates and “fuses” evidence from RNA-seq and whole genome sequencing (WGS) using intron–exon gene structure to identify tumor-specific protein coding gene fusions. Fusion-sq was then applied to the data generated from a pediatric pan-cancer cohort of 128 patients by WGS and RNA sequencing.

**Results:**

In a pediatric pan-cancer cohort of 128 patients, we identified 155 high confidence tumor-specific gene fusions and their underlying structural variants (SVs). This includes all clinically relevant fusions known to be present in this cohort (30 patients). Fusion-sq distinguishes healthy-occurring from tumor-specific fusions and resolves fusions in amplified regions and copy number unstable genomes. A high gene fusion burden is associated with copy number instability. We identified 27 potentially pathogenic fusions involving oncogenes or tumor-suppressor genes characterized by underlying SVs, in some cases leading to expression changes indicative of activating or disruptive effects.

**Conclusions:**

Our results indicate how clinically relevant and potentially pathogenic gene fusions can be identified and their functional effects investigated by combining WGS and RNA-seq. Integrating RNA fusion predictions with underlying SVs advances fusion detection beyond extensive manual filtering. Taken together, we developed a method for identifying candidate gene fusions that is suitable for precision oncology applications. Our method provides multi-omics evidence for assessing the pathogenicity of tumor-specific gene fusions for future clinical decision making.

**Supplementary Information:**

The online version contains supplementary material available at 10.1186/s12885-023-11054-3.

## Background

Gene fusions are important driver mutations in cancer and their accurate detection is essential for diagnosis, treatment selection and understanding disease mechanisms. The fusion of two or more genes through a structural variant (SV) can affect the involved genes directly, but also give rise to a chimeric protein with oncogenic properties [1, 2]. SVs can dysregulate cells in multiple ways, for instance by disrupting genes or by displacing an enhancer resulting in overexpression of oncogenes (e.g. *TLX1/3, NKX2-1*) [3]. Gene fusions are a distinct type of variants characterized by the formation of fusion products and their chimeric transcripts [3]. The contribution of gene fusions to cancer etiology differs per cancer type and they are important for diagnosing pediatric cancers [1, 2]. For example, *KIAA1549–BRAF* fusions in pilocytic astrocytoma and *EWRS1–FLI1* fusions in Ewing sarcoma are prime determinants of these tumor types [2]. Driver fusions also occur in many leukemias [4]. Targeted assays are commonly used in diagnostics as they are reliable, fast and cost-effective, but limited to known partner genes and/or breakpoints [5, 6]. As a result, targeted assays fail to detect some fusions with alternative breakpoints (e.g. *KIAA1549–BRAF)* [7] or which have different partner genes (e.g. fusions involving *TFE3, NUP98, FGFR*) [8–10]. These limitations also make targeted assays unsuitable for discovery of novel gene fusions.

RNA sequencing (RNA-seq) is increasingly applied in both research and diagnostics to detect gene fusions that result in chimeric transcripts. In a pediatric cancer cohort, a 38% increase in diagnostic yield was achieved with RNA-seq compared to traditional diagnostic assays [11]. Here we consider fusions as clinically relevant if they have been published in peer-reviewed journals as associated with specific cancer types and are used for diagnosis, prognosis, or treatment selection. Controlling the false positive rate is a key issue for robust detection of gene fusions based solely on RNA-seq data. Hundreds of chimeric transcripts per sample can be detected by RNA-seq and predicted to reflect gene fusions [12]. Although some have underlying genomic SVs, others result from normal transcription processes such as read-through and intergenic trans-splicing events [13]. Many chimeric transcripts are found in healthy tissue with no known links to malignancy [13, 14]. For example, the translocation resulting in a *PAX3–FOXO1* gene fusion is a driver mutation in alveolar rhabdomyosarcoma, but the chimeric transcript is also transiently expressed in healthy muscle tissue without an underlying SV [13, 14]. Therefore, fusion predictions from RNA-seq require stringent filtering to remove technical artifacts and healthy-occurring chimeric transcripts, as well as to rescue detection of lowly expressed fusions known to be clinically relevant. This introduces bias as the filtering is often done with manually curated inclusion and exclusion lists and limits its use for gene fusion discovery purposes. RNA based ensemble fusion calling methods can reduce the number of false positives, however lowly expressed chimera remain a challenge to detect [15]. Alternatively, identification of the underlying SV can distinguish bonafide tumor-specific gene fusions from artifacts and other chimeric transcripts, as well as provide support to lowly expressed chimeric transcripts without the need for biased manual filtering.

We hypothesize that combining RNA-seq with SVs inferred from whole genome sequencing (WGS) data can help to detect potentially pathogenic gene fusions by identifying breakpoints that support the genomic origin of chimeric transcripts [16] and hence overcome existing limitations of detecting gene fusions based on RNA-seq data alone. By itself, WGS is less suitable for reliable detection of actively transcribed gene fusions as SVs can affect multiple genes and WGS also infers many other non-transcribed variants and similar to RNA-seq, WGS is prone to technical artifacts and false positives [17]. In a heterogeneous cohort of 128 pediatric cancer patients, we identify and interpret tumor-specific gene fusions and resolve the underlying SVs by combined analysis of RNA-seq and WGS data. These SVs are classified as tumor-specific based on analysis of paired tumor/normal WGS samples. We show that combining these orthogonal sequencing methods is promising for genome-wide gene fusion detection as demonstrated for large cancer cohorts [16, 18].

## Materials and methods

### Sample preparation and sequencing

In a pan-cancer cohort of 128 patients diagnosed according to ICD-O-3, RNA and DNA were isolated from fresh frozen tumor tissue and as a matching normal, DNA was isolated from whole blood. Blood and bone marrow samples were enriched for monocytic cells using Ficoll. Total RNA was isolated from tumor samples using the AllPrep DNA/RNA/Protein Mini Kit (QIAGEN) according to standard protocol on the QiaCube (Qiagen). RNA-sequencing (RNA-seq) libraries were generated from 300 ng RNA using the KAPA RNA HyperPrep Kit with RiboErase (Roche) and sequenced with NovaSeq 6000 (2 × 150 bp) (Illumina). DNA was isolated from paired tumor-normal samples also using the AllPrep DNA/RNA/Protein Mini kit. Whole-genome sequencing (WGS) libraries were generated from 150 ng DNA using the KAPA DNA HyperPlus kit and NovaSeq 6000 sequencing platform (Illumina).

### RNA and whole genome sequencing data pre-processing

Pre-processing of RNA-seq and WGS was done with the institute's standardized pipelines implementing GATK 4.0 best practices for variant calling using a wdl and cromwell-based workflow [19, 20]. A minimum median coverage of 25 × for normal and 60 × for tumor WGS data was used, and a minimum 30 million unique reads for RNA data. Further details on quality control are available in Additional file 1: Supplementary Methods and Additional file 2: Quality control metrics. Gene expression was analyzed using featureCounts from Rsubread (version 1.32.4) with Gencode v31 CTAT Oct 2019 annotation.

### Variant calling

Gene fusion predictions were obtained from tumor RNA-seq using STAR-Fusion (version 1.8.0) [21] and GRCh38/Ensembl v97/Gencode v31 CTAT Oct 2019 and FusionCatcher (version 1.33) [22] with Ensembl v97. Structural variants (SVs) were inferred from paired tumor-normal WGS using Manta (version 1.6) [23], DELLY (version 0.8.1) [24] and GRIDSS (version 2.7.2) [25] and the allele fraction was calculated (Supplementary Methods). Fusion-sq was then applied to the RNA based gene fusion predictions and DNA derived SVs (Supplementary Methods). In short, genomic intervals were inferred from intron/exon boundaries to match RNA–DNA breakpoints of respectively gene fusion predictions and SVs.

### Annotation

SVs were annotated with introns based on overlap with canonical transcripts. For each partner gene, a canonical transcript was selected based on stepwise filtering until a single transcript remained: MANE select, the tags "basic, CCDS, APRIS", protein coding, transcript support level and coding sequence length. The transcript annotation was retrieved from Gencode v31 [14]. Annotation for common SVs reported in general population used a 50% reciprocal overlap based on NCBI Curated Common Structural Variants (nstd186) [26], gnomAD Structural Variants (nstd166) [27] from NCBI repository and from DGV (version 2020–02-25) [28] accessed on 2021–03-11. Repeats and segmental duplications annotation was performed using repeats and segmental duplications tracks retrieved from UCSC table browser accessed on 2021–04-20 [29]. Repeats from RepeatMasker were pre-filtered by repeat class (LINE, SINE, LTR) and completeness (< 50 bp of repeats left) to prevent spurious annotations.

To identify whether gene fusions were previously reported in either healthy tissue or cancer samples, we compared our findings to chimeric transcript databases. Fusions were annotated as healthy chimera based on the default annotation from STAR-Fusion [21]. For the annotation of cancer chimera, we used ChimerDB 4.0 (retrieved on 2021–02-17) [30] and the Mitelman database (v20201015, retrieved on 2021–01-07) [1] matching exact gene pairs. Furthermore gene fusions were annotated based on their stable ENSEMBL identifiers and/or gene names against COSMIC (cancer gene census v92) [31], OncoKB (accessed on 2021–04-14) [32] and Grobner et al*.* [33].

## Results

### Fusion-sq: detecting tumor-specific gene fusions with high confidence

**Fig. 1:**
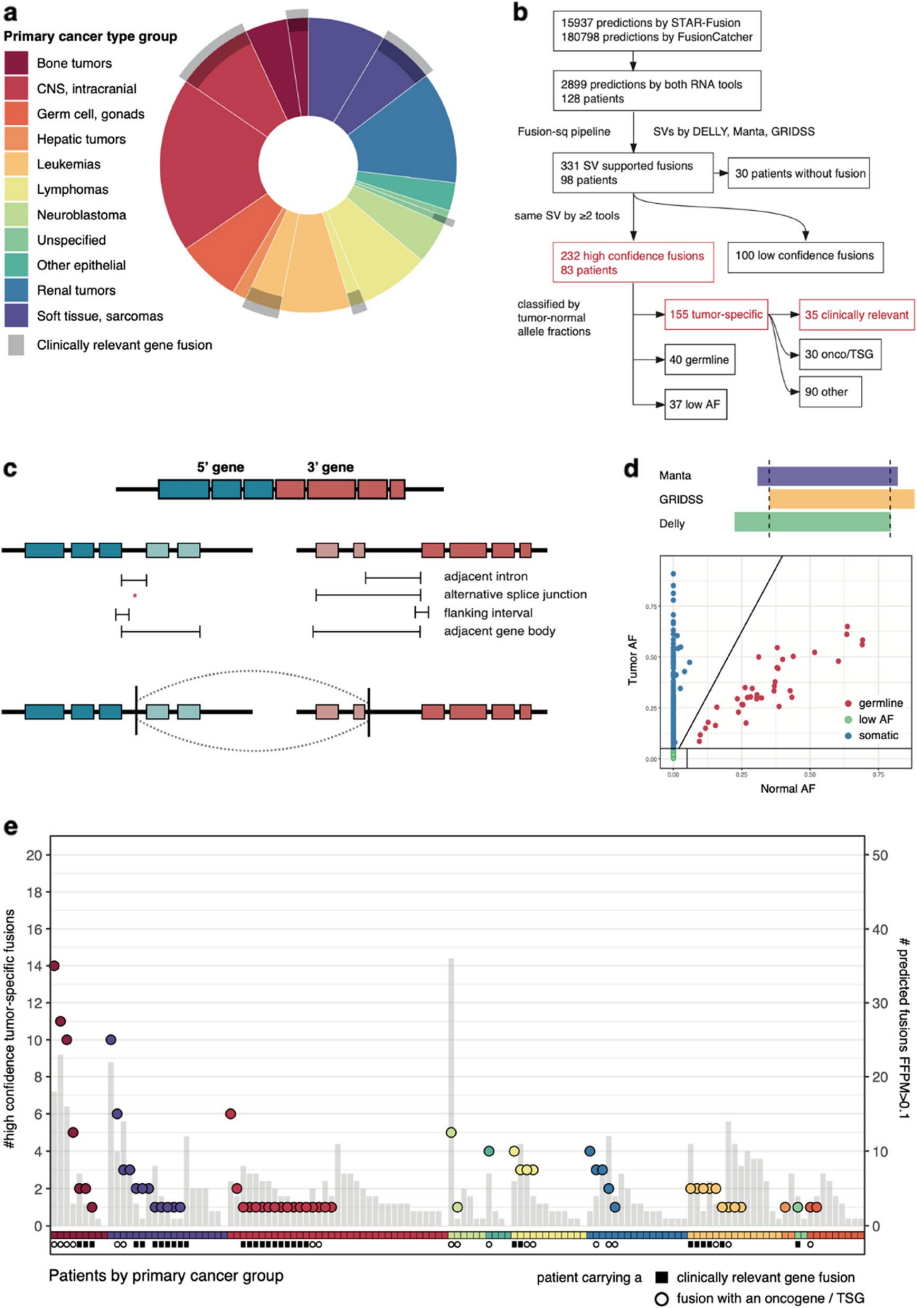
Gene fusion detection in 128 pediatric cancer patients. **a** Distribution of 128 pediatric cancer patients colored according to ICD-O-3 primary group. Gray overlay indicates patients for which a clinically relevant fusion was identified by RNA-seq. **b** Schematic overview of the number of fusions at different steps throughout the Fusion-sq pipeline. Subsets discussed extensively in the main text are highlighted in red and available in Table S1. Note that the 35 clinically relevant gene fusions include reciprocal orientations. In addition, slight differences in numbers with the main text arise from excluding patients without known clinically relevant fusions, e.g. the 27/30 high confidence tumor-specific fusions (hcTSFs) involving oncogenes or tumor suppressor genes (onco/TSG). **c** Schematic overview of the Fusion-sq algorithm to find SVs that support fusion predictions by linking the upstream (5’, blue) and downstream (3’, red) partner genes. First, genomic intervals to match RNA–DNA breakpoints are derived based on the intron–exon gene structure and the RNA breakpoints. Next, DNA breakpoints located in these matching intervals are used to identify SVs that link together the partner genes (Supplementary Methods). **d** Upper panel: schematic representation of high confidence fusion detection based on SVs identified by two or more tools (> 50% reciprocal overlap). Lower panel: scatter plot of tumor and normal allele fraction (AF), resulting in classification of fusions in tumor-specific (blue), likely germline (red) and low AF (green). **e** Number of fusions in individual patients: hcTSFs (circles, grouped and color-coded by primary cancer type group) and fusion predictions by both RNA fusion tools with more than 0.1 fusion fragments per million total RNA-seq fragments (FFPM) (gray bars). Annotation indicates whether patients carry a gene fusion that is clinically relevant (black square) or involves an oncogene or tumor suppressor gene (open circle)

To resolve gene fusions and investigate their relevance to pediatric cancer, we combined RNA-seq and paired tumor/normal WGS samples of 128 patients across 53 pediatric cancer types (Fig. 1a). Known clinically relevant fusions were identified with RNA-seq in 30 patients (Fig. 1a, gray overlay). We investigated combining WGS structural variant analysis with RNA-seq data to increase detection specificity and identify potentially pathogenic gene fusions in the remainder of the cohort. Hereto, we developed Fusion-sq which integrates (“fuses”) predicted gene fusions from RNA-seq data with SVs from WGS data (Methods, Fig. 1b,c). For every predicted gene fusion, Fusion-sq first derives genomic intervals to match RNA and DNA breakpoints based on intron–exon gene structure. Next, DNA breakpoints falling within these intervals are used to identify SVs that link the fusion 5’ and 3’ partner genes. To optimize both recall and precision, gene fusion predictions from STAR-Fusion and FusionCatcher are integrated with SVs detected by Manta [23], DELLY [24] and GRIDSS [25]. We selected high confidence fusions that are 1) predicted by both RNA gene fusion tools and 2) supported by SVs detected by at least two callers (Fig. 1b, d, Additional file 1: Figure S1). Fusions are then classified as tumor-specific, (likely) germline or low allele fraction (AF) based on the SV’s variant supporting reads in the paired tumor/normal samples, as reflected in the AFs of SVs in both samples (Fig. 1b,d). In total, 2899 fusions were predicted in 128 patients using RNA-seq data alone (Fig. 1b). By combining these fusion predictions with WGS data, Fusion-sq identified 232 high confidence fusions in 83 patients (Fig. 1b, e). To investigate potentially pathogenic gene fusions, we further analyzed the 155 high confidence tumor-specific fusions (hcTSF) by combining RNA evidence with properties of the underlying SVs, such as SV type and AF.

### All clinically relevant fusions match to tumor-specific SVs

**Fig. 2:**
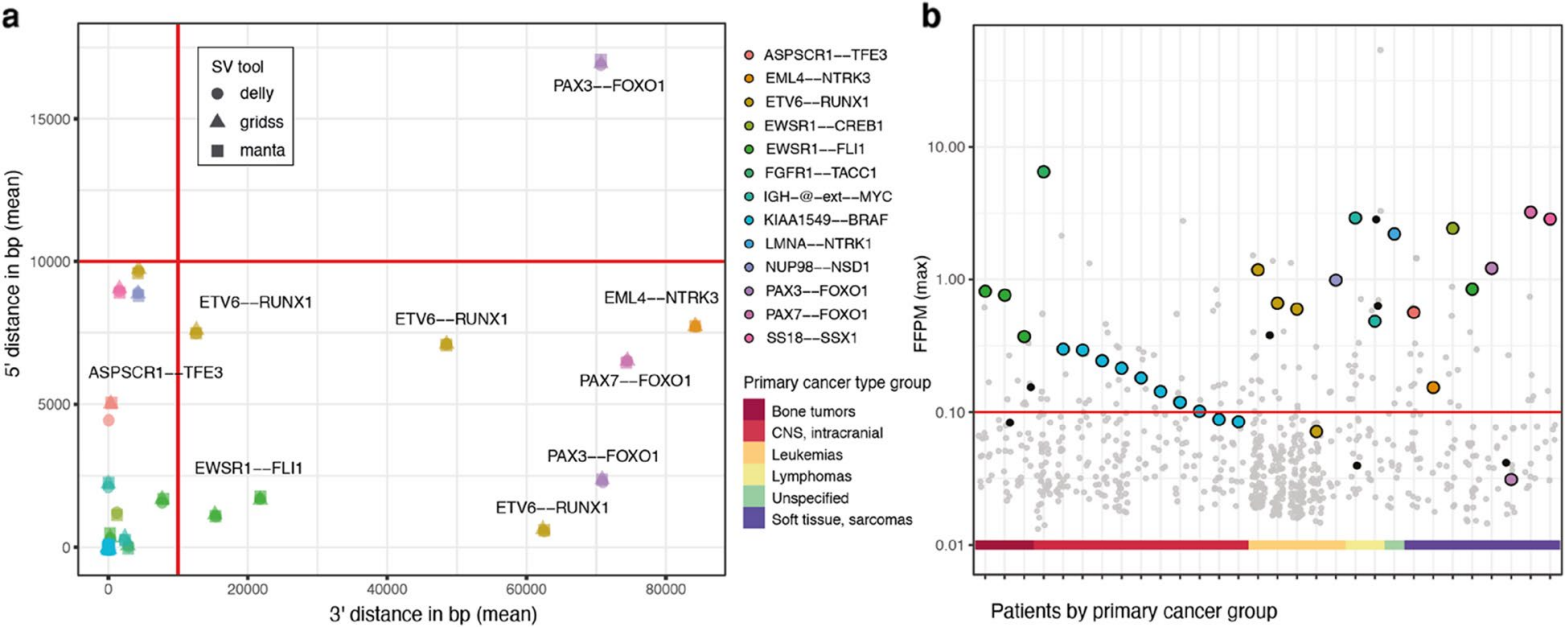
Tumor-specific SVs resolve clinically relevant fusions with high confidence. **a** Distance between RNA breakpoints and matching SVs as resolved by DELLY (circle), Manta (square) and GRIDSS (triangle) for the 5' and 3' partner genes in 30 patients carrying clinically relevant fusions. Colors represent specific gene fusions. Red lines indicate 10 kb fixed-size matching intervals and would fail to match SVs for nine gene fusions (labeled). Note that all clinically relevant fusions are detected by at least two SV tools at nucleotide resolution (< 10 bp, overlapping symbols) except for *ASPSCR1–TFE3*. **b** Gene fusion predictions supported by both RNA fusion tools (all circles, same patients as in **a**) with their read support in fusion fragments per million total RNA-seq fragments (FFPM). The red line indicates the default cutoff below which fusions are usually discarded (low expression FFPM < 0.1). Supporting high confidence tumor-specific SVs were identified for all clinically relevant fusions (colored circles; colors same as in **a**) and seven additional fusions (black) of unknown significance, but not for the remaining RNA-only fusion predictions (gray). Colored bar at the x-axis indicates the primary cancer type group

We first focused on the subset of 30 patients known to carry a clinically relevant gene fusion. In all patients, we resolved the tumor-specific SVs for the predicted gene fusions (Table S1). Validation with traditional diagnostic assays such as FISH, karyotyping, SNP array and RT-PCR was conducted for 28 fusions (Table S2). To better understand gene fusions at the genomic level, we investigated the underlying SVs supporting the chimeric transcripts. For all patients but one, the associated duplications (DUP), deletions (DEL) and inversions (INV) were almost identical (> 99% overlap) and breakpoints of interchromosomal translocations (CTX) were resolved within 10 base pairs (bp) showing a high agreement between SV callers (Fig. 2a). Identification of the underlying SV also helped to reconcile variability in RNA-based predictions as shown by the SVs matching to multiple predictions (Table S1). The distances between RNA breakpoints from the chimeric transcripts and corresponding DNA breakpoints from the underlying SVs are highly variable amongst gene fusions and patients (Fig. 2a). Notably, for nine patients the distance between corresponding RNA–DNA breakpoints is larger than 10 kilobase pairs (kb) (Fig. 2a, red lines), illustrating the advantage of using intron–exon gene structure to define genomic intervals for matching chimeric transcripts with SVs.

One clinically relevant fusion that proved difficult to resolve was *ASPSCR1–TFE3,* as the underlying translocation t(7;X) was identified differently by Manta, DELLY and GRIDSS (Table S1). Manta resolved it as a composite fusion of a CTX + INV in chrX, whilst GRIDSS and DELLY had unusually broad CTX footprints of 393 bp and 1181 bp respectively. The associated SV breakpoints of all three tools overlapped SINE elements, which may indicate difficulties in resolving this gene fusion to base pair accuracy in DNA. Despite this potentially complex variant, this translocation could still be mapped to the *ASPSCR1–TFE3* chimeric transcript by Fusion-sq.

Simple SV events underlie the remaining (n = 29) clinically relevant fusions [34] and represent all major SV types, such as deletions, duplications, inversions and translocations. As expected, interchromosomal translocations (CTX, n = 18) were the most common SV type underlying clinically relevant gene fusions [1]. In some cases, these translocations support both the canonical and reciprocal transcripts. We identified a reciprocal gene fusion product for three out of four patients with an *ETV6–RUNX1* fusion, both patients with an *IGH–MYC* fusion, and for one out of four patients with an *EWSR1–FLI1* fusion. In nine patients, we identified duplications resulting in *KIAA1549–BRAF* fusions. Interestingly, the *KIAA1549–BRAF* fusion of patient M218AAA was identified as an inversion by all three SV callers, and it is likely an inverted duplication as read depth is also increased (+ 0.42 copy number log2 fold change, CN l2fc). The two remaining clinically relevant fusions are *FGFR1–TACC1* caused by a 420 kb INV, and *LMNA–NTRK1* caused by a 740 kb DEL, showing that a variety of SV types can result in clinically relevant fusion events.

To reduce false positives in RNA-seq fusion detection, filtering based on read support for chimeric transcripts is often implemented with a default minimum of 0.1 fusion fragments per million total RNA-seq fragments (FFPM) [2, 35]. However, by relying only on RNA evidence, four clinically relevant fusions would be missed due to their low expression (Fig. 2b, Table S1), two *KIAA1549–BRAF* fusions (0.08–0.09 FFPM), one *ETV6–RUNX1* (0.07 FFPM) and one *PAX3–FOXO1* (0.03 FFPM). These lowly expressed fusions can be discerned from false positives by integration with WGS. In total, 715 fusions were predicted by both STAR-Fusion and FusionCatcher in these 30 patients alone, of which 147 passed the read support threshold (FFPM > 0.1). In contrast, for 24 patients their clinically relevant gene fusions are the only hcTSFs indicating a high specificity of Fusion-sq. In the remaining six patients, Fusion-sq resolved an additional seven hcTSFs with similar support but of unknown clinical relevance (Fig. 2b, black dots). This shows that integration of RNA-seq and WGS by Fusion-sq can accurately resolve tumor-specific fusions, effectively removing the need for extensive manual filtering to select fusions for follow-up.

### Underlying SVs distinguish tumor-specific fusions from healthy chimera

The 232 high confidence fusions (hcFs) identified in 83 patients were classified as tumor-specific, germline or low AF (Fig. 1b). Both the clinically relevant and additionally detected hcTSFs have a higher tumor AF relative to the normal AF, whereas germline fusions and low AF fusions show similar tumor and normal AFs (Additional file 1: Figure S2). While it is counter-intuitive to have high confidence variants with these low AFs, we reasoned that a high number of variant and reference reads could explain this. Indeed, 81% of these low AF hcFs originated from amplified regions (CN l2fc > 1.58). Next, we evaluated the efficacy of identifying hcTSFs by 1) assessing the underlying SV properties, 2) annotating with databases of chimera and SVs, and 3) annotating with cancer-related genes.

**Fig. 3:**
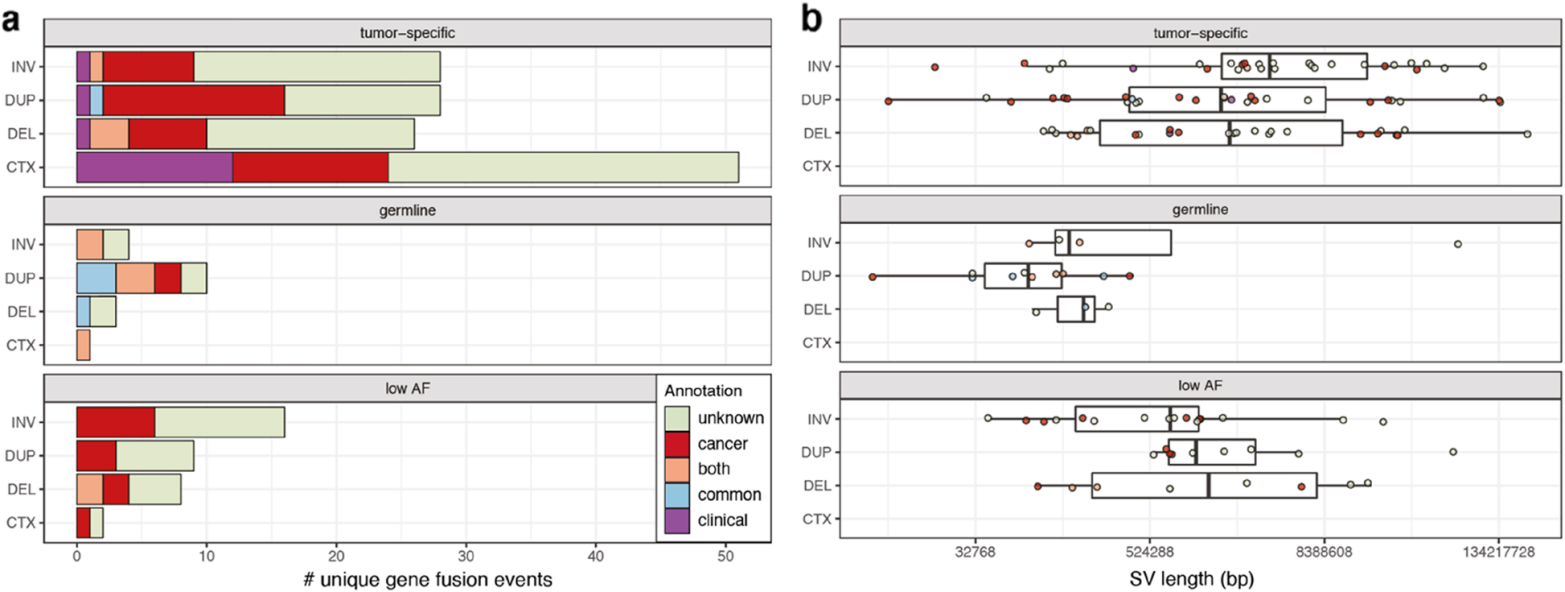
Underlying SVs distinguish tumor-specific fusions from healthy chimera. Number of (**a**) and average SV length in base pairs (bp) for (**b**) distinct high confidence fusions categorized either as tumor-specific (upper panel), likely germline (middle panel) and low AF (lower panel). Each category is further subdivided according to SV type: inversion (INV), duplication (DUP), deletion (DEL) or interchromosomal translocation (CTX). Fusions are colored based on known clinical relevance (clinical; purple), involving a cancer-related gene or cancer chimera (cancer; red), population SV or healthy chimera (common; blue), or both cancer and common (both; orange)

We then investigated whether the underlying SVs of additionally detected gene fusions resemble those of known clinically relevant fusions. Hereto, we mapped the high confidence fusions resolved in individual patients to distinct fusions to account for recurrent fusions. In total the 232 hcFs mapped to 189 distinct fusions of which 134 tumor-specific, 18 germline and 37 low AF (Table S3, Additional file 1: Figure S3). Like the clinically relevant fusions, interchromosomal translocations (CTX, 39 of 119) are the most common SV type underlying the additionally identified tumor-specific fusions. The remaining tumor-specific intrachromosomal SVs are distributed over DUP, DEL and INV (Fig. 3a). In contrast, germline fusions are depleted in CTX events and generally caused by shorter intrachromosomal SVs. The tumor-specific SVs are approximately 3.5 × larger than germline SVs (Fig. 3b). Only a single germline fusion (*CCDC32–CBX3*) has an underlying CTX. *CCDC32–CBX3* is a known healthy chimera and the SV breakpoints overlap ALU repeat elements which indicates either potential mapping difficulties or a mechanism involved in forming this event. In conclusion, these results show that the types and sizes of SVs underlying tumor-specific gene fusions are distinct from germline events and resemble the SVs of known clinically relevant fusions.

Secondly, to compare hcTSFs with population variants, the gene fusions were annotated with databases cataloging healthy chimeric transcripts or databases of SVs occurring in the general population as normal variation (Table S3), such as NCBI Common SV database [26], gnomAD [27] and DGV [28]high confidence fusions, ten are flagged as a healthy chimera by the gene fusion caller, of which seven were also classified as germline fusions. Further comparison of SVs underlying fusions with SVs occurring in the general population showed overlap for eight germline fusions and five tumor-specific fusions. Overall, 56% of the distinct germline hcFs either overlap a population SV or occur in a healthy chimera database, compared to only 4% of tumor-specific fusions (Fig. 3a). These results indicate that the hcTSFs are depleted for germline population variants.

Finally, to identify potentially pathogenic fusions, we compared the results of Fusion-sq with chimera previously detected in cancer and annotated fusions with cancer-related genes. 44 of the 189 distinct hcFs are present in ChimerDB [30] or the Mitelman database [1]. Most of these are classified as tumor-specific (28), but also germline (8) and low AF (8) fusions occur in these databases. Six of the eight germline fusions annotated as cancer chimera are also annotated as either healthy chimera or have underlying intrachromosomal SVs that overlap with general population SVs (Fig. 3a). This corresponds to previous observations that cancer chimera databases can include passenger fusions [36, 37]. After accounting for recurrence, 128 tumor-specific fusions were identified which have no evidence of occurring in the normal population. Next, we annotated the fusion partner genes as proto-oncogene or tumor-suppressor gene (TSG). This further substantiated the classification into tumor-specific and germline fusions based on their underlying SVs, since germline hcFs do not include oncogenes or TSGs (Fig. 3a). In addition to the known clinically relevant fusions, we identified 27 distinct hcTSFs involving oncogenes and/or TSGs that warrant further investigation of their underlying SVs and functional effects.

### Resolving tumor-specific fusions in individual genomes

**Fig. 4:**
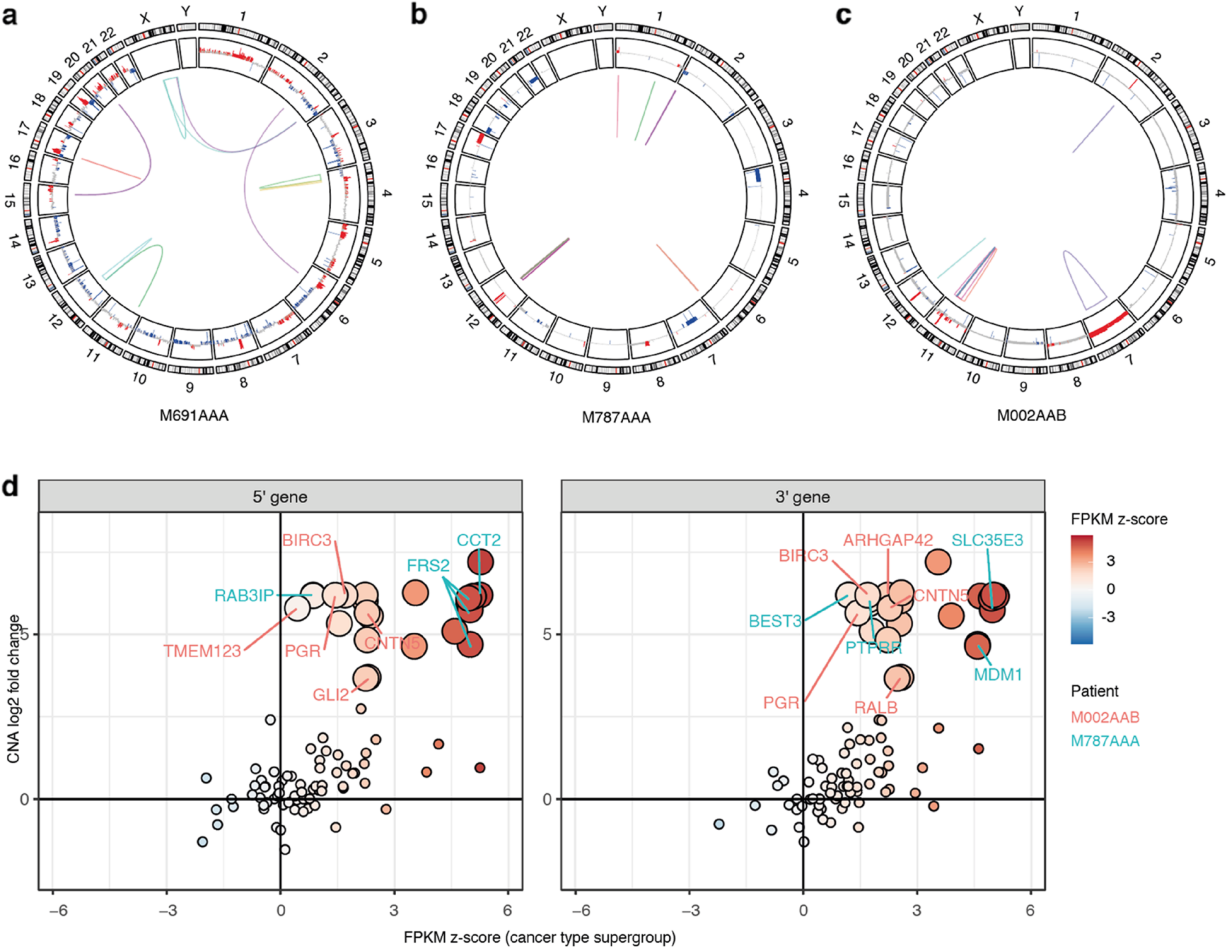
Resolving tumor-specific fusions in patients with high copy number instability and focal amplifications. Circos plots of osteosarcoma patient M691AAA (**a**), neuroblastoma patient M787AAA (**b**) and embryonal rhabdomyosarcoma patient M002AAB (**c**). The plots are annotated with high confidence tumor-specific gene fusions (multi-colored links) and copy number gains (red) and losses (blue). **d** Relationship between copy number alterations and gene expression changes of the 5' (left) and 3' (right) partner genes. Gene expression in fragments per kilobase of transcript per million mapped reads relative to the cancer type supergroup (FPKM z-score, color scale displayed on the right) and copy number log2 fold (CN l2fc) change relative to each tumor’s read depth baseline. Genes located inside amplified regions (CN l2fc > 1.58) are displayed with larger circles. Cancer-related genes are labeled according to their patient of origin

Focusing on patients without a known clinically relevant fusion, we identified 113 hcTSFs of unknown significance to investigate further. These fusions are detected in 32 patients across different cancer types. Generally, each individual patient carries few to none hcTSFs (Fig. 1e), whereas a high burden of gene fusions is associated with copy number instability (Fig. 4. Additional file 1: Figure S4). High fusion burden (> = 5 hcFs, ~ 95th percentile) is associated with a high fraction of genome altered (FGA) by copy number alterations (CNAs) (median 63% vs 3.8%, mean 48% vs 11%, wilcox *p* < 0.01) in tumor types prone to copy number instability such as osteosarcoma (4x), embryonal sarcoma, embryonal rhabdomyosarcoma, neuroblastoma and ependymoma [33].

To further investigate this association between copy number instability and high fusion burden, we closely studied four osteosarcoma patients carrying *TP53* and *ATRX* fusions (Additional file 1: Figure S5). These patients’ tumors have many dispersed CNAs (median FGA 67%) and many hcFs distributed across their genomes (range 5–15, Fig. 1e, Additional file 1: Figure S5a). In three of these osteosarcoma patients, we resolved fusions involving the first exon of *TP53* and in each case a different 3’ partner gene resulting from an inversion and two translocations; t(17;6), t(17;20) (Additional file 1: Figure S5b) (Table S1). Translocations with the first exon of *TP53* have previously been identified as cancer driver events in osteosarcoma [38]. Therefore, these fusions are potentially also pathogenic in these patients, especially since a driver mutation had yet to be identified for these tumors. Apart from the *TP53* gene fusions and associated SVs, we did not observe additional CN losses or deleterious SNVs in the *TP53* loci of these patients. In the fourth osteosarcoma patient (M691AAA, Fig. 4a), we detected an *ATRX* fusion which has been suggested previously as a potential driver mutation for osteosarcoma [38]. For the *ATRX* fusion, gene expression concomitantly was reduced relative to the cancer type supergroup (-3.3 z-score of the fragments per kilobase of transcript per million mapped reads (FPKM) (zfpkm), *p* < 0.01). Similarly, the group of patients with a *TP53* fusion showed reduced expression relative to the cancer type supergroup (0.88 vs 2.0 log2 FPKM, *p* < 0.05) (Additional file 1: Figure S5c). However, this was not clear for the individual patients, illustrating that the underlying SV provides additional evidence for a disruptive SV event that could not be easily derived from RNA-seq alone.

Multiple fusions originating from highly amplified regions were resolved in two patients with neuroblastoma (M787AAA, Fig. 4b) and embryonal rhabdomyosarcoma (M002AAB, Fig. 4c). The neuroblastoma patient has a focal amplification in chromosome 12q13-15 involving the oncogenes *MDM2* and *CDK4* (4–6 copy number log2 fold change, CN l2fc). In this region, we identified 18 high confidence, low AF gene fusions. Including fusions previously identified as cancer-related chimera (i.e. *FRS2–MDM1, FRS2–PTPRR, CCT2–BEST3, RAB3IP–BEST3*) of which both partner genes are overexpressed due to the amplification (Fig. 4d). Similarly, patient M002AAB carries 11 fusions originating from a focal amplification in chr11q22 (5–7 CN l2fc) which are detected with high confidence but some with a low AF. Here also, the fusion partner genes are overexpressed which is consistent with the amplification (Fig. 4d). Although chr11q22 amplification itself is not known to be clinically relevant, we resolved multiple fusion combinations with oncogenes *BIRC3*, *PGR* and in particular *YAP1*. *YAP1–CFAP300* involves exons 1–5 of the *YAP1* oncogene which is highly amplified (7.2 CN l2fc) and overexpressed (5.3 zfpkm, *p* < 0.1). Also, this exon 1–5 fragment has previously been identified as pathogenic when fused to other 3’ partner genes by activating the TEAD pathway [39, 40].

These case studies illustrate that Fusion-sq can confidently resolve fusions in unstable genomes with a high FGA or complex alterations. For some tumor-specific fusions, the underlying SVs are potentially pathogenic (e.g. the *TP53* and *ATRX* fusions) while in other cases the fusions seem to be the result of copy number instability. In addition, gene fusions in amplified regions can exhibit high expression of their partner genes due to the underlying CN gain. Therefore, characteristics of underlying SVs are key for interpreting potential functional effects of individual gene fusions, especially in patients with unstable genomes.

### Tumor-specific fusions affecting gene expression

Gene fusions can activate oncogenes or disrupt tumor-suppressor genes (TSG) and the resulting pathogenic effects can be reflected in dysregulation of gene expression. To identify potentially pathogenic fusions, we assessed the functional effects of the 27 distinct hcTSFs involving an oncogene or TSG (in **bold**) (Table S1, Additional file 1: Supplementary Results and Figures S6—S10). Their SVs represent all the simple SV types: CTX (10), DUP (7), DEL (5) and INV (5). As a proxy for functional effect, we combined expression data, underlying SVs and gene annotation.

**Fig. 5:**
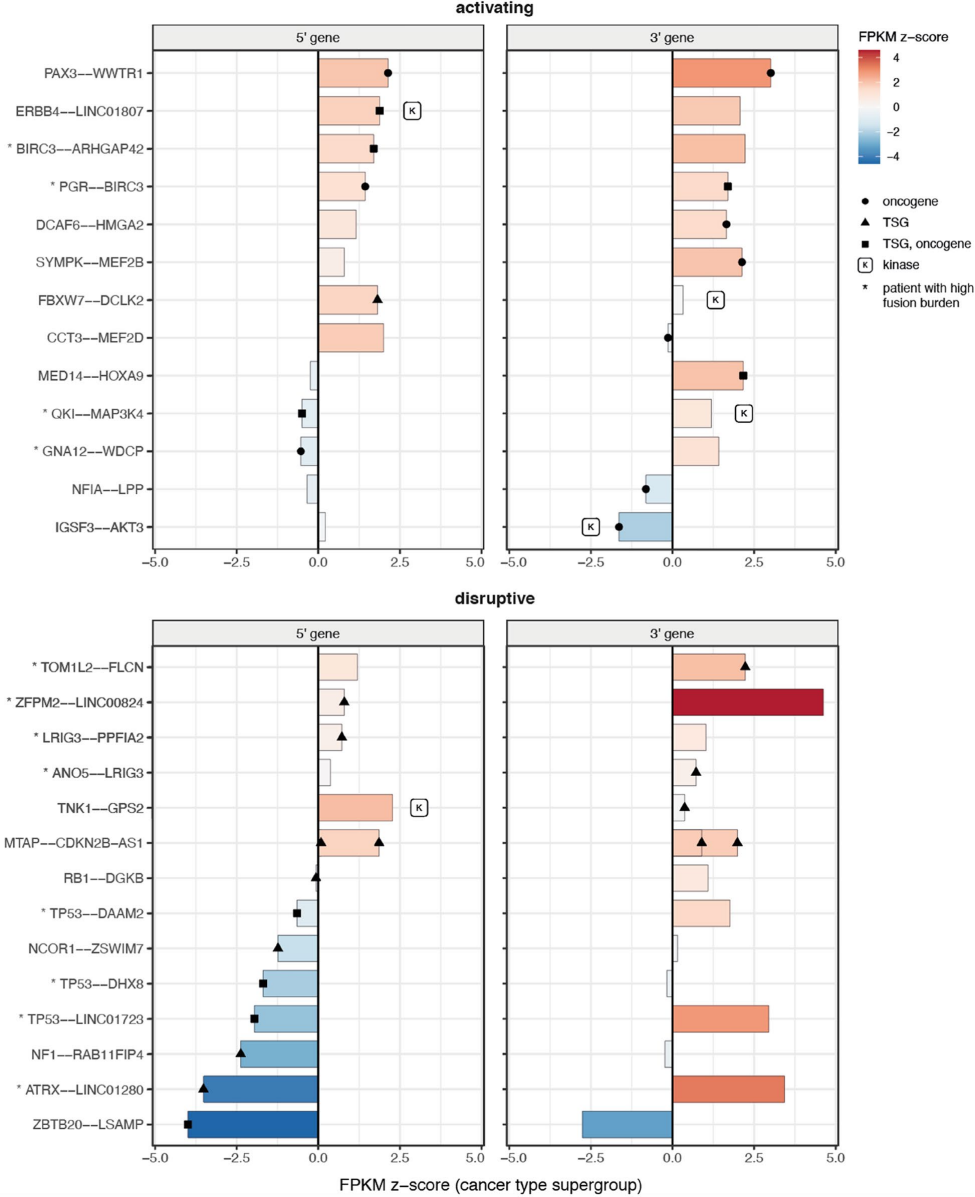
Fusion partner gene expression can indicate oncogene activation or tumor-suppressor gene disruption. Gene expression changes for the 5' (left) and 3' (right) partner genes of tumor-specific gene fusions involving oncogenes and/or tumor-suppressor genes (TSG). Changes are relative to the cancer type supergroup (FPKM z-score; color scale on the right). Individual 5' or 3' genes are marked as either oncogene (circle), TSG (triangle), both TSG and oncogene (square) or kinase (k). Fusions were divided into ‘activating’ or ‘disruptive’ functional effect categories based on partner gene annotation, and marked with an asterisk if they originate from patients with a high gene fusion burden

Overexpression of oncogenes resulting from gene fusions is often suggested as an activation mechanism [2]. However, we did not observe an enrichment of oncogenes amongst overexpressed fusion partner genes (> 1.96 zfpkm) compared to the cancer type supergroup or the full cohort. Instead, our data suggests an association with copy number gain (CN l2fc > 0.58) irrespective of gene annotation, as reflected by approximately half of the fusions with CN gain found to be overexpressed (24 of 42). We identified fusions in individual patients for which the 3’ oncogenes are significantly overexpressed relative to the cancer type supergroup (*p* < 0.1): ***PAX3–WWTR1*** (2.1 and 3.0 zfpkm) and *SYMPK–MEF2B* (2.1 zfpkm) (Fig. 5). In addition, we identified a *MED14–HOXA9* fusion and associated overexpression of *HOXA9* (2.2 zfpkm, *p* = 0.15) in a pre-T-cell lymphoblastic leukemia patient (M385AAA) (Additional file 1: Supplementary Results, Figure S6 and S7).

Gene fusions can also be pathogenic through activation of kinases due to e.g. loss of an auto-inhibitory domain or increased dimerization. We identified eight hcTSFs involving kinases, of which three in patients with unstable genomes (*QKI–MAPK3K4, PRKD3–LTBP1, STK40–OSCP1*) and three fusions (*ERBB4–LINC01807, FBXW7–DCLK2* and *IGSF3–AKT3)* with unknown relevance since the kinase domains are not part of the chimeric product. However, two fusions resulted in a chimera containing the kinase domains, *MEF2A–IGF1R* and *TNK1–GPS2*. The *TNK1–GPS2* fusion was found in a patient with anaplastic large cell lymphoma resulting from a 74 kb inversion (0.40 tumor AF). Resolving the underlying SV confirmed the presence of this gene fusion which was predicted with different breakpoints and in reciprocal orientations by the two RNA-seq tools (Additional file 1: Supplementary Results, Figure S6).

Finally, we identified three gene fusions involving tumor-suppressor genes (TSGs) which showed a significant decrease in gene expression (*p* < 0.1) relative to the cancer type supergroup: ***ZBTB20–LSAMP*** (-4.0 zfpkm, *p* < 0.05), ***NF1–RAB11FIP4*** (-2.4 zfpkm) and the previously mentioned ***ATRX–LINC01280*** (-3.5 zfpkm) in a patient with osteosarcoma (Additional file 1: Supplementary results, Figure S6).

These examples illustrate that combining WGS structural variant identification with RNA-seq based gene fusions results in the identification of novel high confidence gene fusions with strong biological relevance.

## Discussion

Discovery of novel driver gene fusions in pediatric cancer is limited by a lack of methods for genome-wide unbiased detection. To systematically discover tumor-specific gene fusions, we developed Fusion-sq which integrates chimeric transcripts from RNA-seq with SVs from WGS using intron–exon gene structure. Previous studies combining RNA-seq and WGS data for gene fusion detection vary in validation rates but also in the detection and integration methods used [16, 37, 41, 42]. We identified supporting SVs for ~ 11% of gene fusions predicted by both RNA-seq tools, similar to the previously reported 91% false positive rate [41]. In contrast, the PCAWG transcriptomics group identified supporting SVs for 82% of RNA alterations [16]. This difference is likely due to stringent pre-filtering of predictions and lenient RNA–DNA breakpoint matching with 500 kb intervals. We identified SVs that precisely support chimeric transcripts and abstained from expression-based filtering, which would have excluded four clinically relevant fusions. The recently published tool MAVIS also combines RNA-seq data with SVs to identify gene fusions and takes intron–exon gene structure into consideration, and additionally contains an assembly step to identify breakpoint sequences [42, 43]. In contrast, Fusion-sq uses a hierarchy of matching intervals for RNA and DNA breakpoints that allows for reporting fusions with different levels of agreement between these data types (Supplementary Methods). This can rescue fusions where it is challenging to match the evidence from RNA and DNA, such as for the *ASPSCR1–TFE3* fusion with breakpoints in SINE repeats. Other pediatric cancer studies focusing on precision oncology rely heavily on experts to perform the data-integration manually [18, 44] thereby limiting its applications for candidate discovery.

Recurrence is often used to distinguish potentially pathogenic fusions from passenger fusions [16, 18, 42]. This approach yielded little results in our pediatric pan-cancer cohort, likely due to the cohort size, high heterogeneity of cancer types and low mutation burden of pediatric cancers. In addition to the known clinically relevant fusions, the only other recurrent fusion identified was *MTAP–CDKN2B-AS1.* Yet, we identified multiple potentially pathogenic gene fusions in individual patients by leveraging SV properties and gene expression data. Analyzing the underlying SVs of gene fusions can discern tumor-specific fusions from likely passenger fusions that are the result of normal transcription processes, germline SVs or are related to copy number instability. Consistent with adult cancers [1, 37], a high gene fusion burden is associated with a high FGA, or unstable regions such as focal amplifications. Despite recurrence being an important criterion for defining clinically relevant fusions, our results indicate that alternative strategies to recurrence can identify potentially pathogenic fusions as candidates for follow-up investigation.

Detection of gene fusion chimeric transcripts using RNA-seq data is limited to actively transcribed genes, and fusions with non-coding elements may be missed. In addition to protein-coding fusion products, SVs that displace enhancers can cause unusually high expression of oncogenes [3]. However, these “enhancer hijacking” events fall outside the scope of this study as they lack chimeric transcript evidence. For example, an *IGH–MYC* translocation was identified with FISH in patient M879AAA and in the WGS data we also identified the underlying reciprocal translocation from the *IGH* locus to ~ 200 kb upstream of *MYC*. Also chimera resulting from intergenic fusions can be missed, since they arise from SVs followed by additional splicing alterations and may not have SV breakpoints corresponding to their chimeric transcript [45]. Further development of gene fusion detection algorithms could result in improved identification of events not resulting in a chimeric transcript. In the near future, long-read WGS could aid in the detection of complex SVs and long-read RNA sequencing is promising for detecting full-length isoforms of gene fusions [46].

Requiring orthogonal support from WGS is effective in filtering potential false positives from RNA-seq, and chimeric transcripts without underlying genomic mutations that result from normal transcription processes (i.e. read-through events or cis/trans splicing) [13, 36]. Many of these RNA-only chimeric transcripts also occur in healthy tissues and are less likely to have a pathogenic effect compared to fusions caused by tumor-specific SVs [13, 14]. Of the few germline hcFs we resolved, the SVs are distinct from those underlying the tumor-specific fusions (Fig. 3). Whilst both gene fusion and SV detection are prone to false positives and false negatives, requiring support from RNA and DNA greatly reduces the risk of false positives. Validation with targeted assays was available for most clinically relevant fusions and these were in agreement with the results from Fusion-sq (Table S2). Although sufficient quality RNA and tumor-normal paired WGS can be difficult to obtain for certain types of cancer, we could confidently detect lowly expressed gene fusions such as *KIAA1549–BRAF* and fusions in samples with low tumor cell percentages (30–40%) (Table S2). We have previously shown that gene fusion detection with RNA-seq achieves a higher sensitivity than targeted assays [11] and our results here indicate that WGS data is promising for improving specificity.

Overall, we identified 27 distinct potentially pathogenic gene fusions in 19 patients that involve oncogenes or TSGs and display similar characteristics to fusions that have previously been linked to tumor etiology. For some patients, these candidate fusions add to the list of variants of unknown significance which have been identified in their tumor genomes, but for others the identified gene fusion presents a strong candidate for follow-up studies (Additional file 1: Supplementary Results). These candidate pathogenic fusions include events which potentially activate known oncogenes, transcription factors and kinases. As well as gene fusions that disrupt TSGs. While it is known that gene fusions can result in activation of oncogenes, less is known about fusions involving TSGs [2, 47]. Fusions that disrupt TSGs can be promiscuous in both their partner genes and breakpoints making them difficult to detect with targeted assays [7, 8]. Demonstrated by the three *TP53* fusions within this cohort each with different partner genes. These results further emphasize the power of genome wide sequencing for the identification of individually rare, but mechanistically common gene fusion events.

It is difficult to assess the pathogenicity of individual fusions in patients with copy number unstable genomes and many gene fusions. For example, the co-amplification and resulting overexpression of *MDM2/CDK4/FRS2* in neuroblastoma patient M787AAA is clinically relevant [48], and the fusions originating from this amplification are more likely to be passenger events [37, 49]. Resolving the underlying SVs and copy number alterations (CNAs) can distinguish expression changes due to “catastrophic” genomic events from pathogenic fusions where 3’ (onco)genes are upregulated due to fusion with an active promoter. We did not observe strong cohort-level trends of gene fusions resulting in 3’ oncogene overexpression, likely because of the highly specific associations between oncogenes and certain pediatric cancer types. Instead, we found an association between CN gain and fusion partner gene overexpression, consistent with observations in adult cancers that CNAs are the main contributing factor to gene expression changes [16].

## Conclusion

To increase our understanding of pediatric cancer, new approaches should be developed to identify novel driver gene fusions. Here, we identified tumor-specific gene fusions with high confidence by combining chimeric transcripts from RNA-seq and SVs from WGS data. Resolving the underlying SVs enables confident detection of known clinically relevant fusions, as well as discovery of potentially pathogenic fusions by distinguishing them from artifacts and healthy-occurring events.

SVs can aid clinical decision making through the selection of tumor-specific fusions for targeted therapies and aid minimal residual disease monitoring by providing allele fractions and exact breakpoints. We identified 27 potentially pathogenic tumor-specific gene fusions involving oncogenes and tumor-suppressor genes and demonstrated how these events can be linked to gene expression changes. Rare gene fusions are difficult to interpret, without recurrence, they require further investigation into biological mechanisms or pathways. The approach used in this study is not only useful for pediatric cancer but can also be applied in adult cancer for identifying candidate pathogenic fusions. Overall, we show the power of integrating RNA-seq gene fusion predictions with WGS structural variants, which aids discovery and interpretation of pathogenic fusions for precision oncology applications.

## Supplementary Information

**Additional file 1: **Supplementary material. Supplementary Methods. Supplementary Results. **Figure S1.** Gene fusions supported by one or more SV tools. **Figure S2.** Allele fraction of distinct fusions per SV type. **Figure S3.** Filtering predicted gene fusions to a high confidence subset. **Figure S4.** High gene fusion burden is associated with copy number instability. **Figure S5.** Osteosarcomas with TP53 gene fusion. **Figure S6.** Potentially pathogenic gene fusion candidates in individual patients. **Figure S7.** HOXA9 gene expression and gene fusion status. **Figure S8.** Association between ZBTB20 gene expression and survival. **Figure S9.** Co-occurring fusions and SNVs indicative of TSG disruption. **Figure S10.** MTAP--CDKN2B-AS1 fusions and associated expression changes of CDKNB-AS1 and CDKN2A. References for supplementary methods and results.

**Additional file 2.** Quality control metrics. Data quality metrics for the WGS and RNA data used in this study: median and mean coverage, percentage of duplicates and read counts.

**Additional file 3.** Column descriptions for tables S1 and S2.

**Additional file 4:** **Table S1.** High confidence tumor-specific gene fusions. Gene fusions for which underlying SVs are resolved with high confidence as tumor-specific or low AF variants. Also included are the gene fusions caused by a composite SV detected by at least two tools (e.g. ASPSCR1--TFE3). See Additional file 3 for column descriptions.

**Additional file 5:** **Table S2.** Validation by targeted assays for clinically relevant gene fusions. For 28 clinically relevant fusions targeted assays were conducted validating the presence of the fusion. Abbreviations: fluorescence in situ hybridization (FISH), single nucleotide polymorphism (SNP) array, reverse transcription polymerase chain reaction (RT-PCR), and immunohistochemistry (IHC).

**Additional file 6:** **Table S3.** Distinct high confidence gene fusions. Gene fusions from Table S1 mapped to distinct fusions such that fusions occurring in multiple patients are merged and listed only once.

## Data Availability

The WGS and RNA-seq datasets supporting the conclusions of this article are available in the EGA repository, dataset accession numbers EGAD00001008152. Code for fusion-sq is available through https://github.com/princessmaximacenter/fusion-sq. Extended data tables are included as supplemental data.

## Abbreviations

AF: Allele fraction
bp: Base pairs
CN l2fc: Copy number log2 fold change
CNA: Copy number alteration
CTX: Interchromosomal translocations
DEL: Deletion
DUP: Duplication
FFPM: Fusion fragments per million total RNA-seq fragments
FGA: Fraction of genome altered by copy number alterations
FPKM: Fragments per kilobase of transcript per million mapped reads
hcFs: (Set of) high confidence fusions
hcTSFs: (Set of) high confidence tumor-specific fusions
INV: Inversion
kb: Kilobase pairs
RNA-seq: RNA sequencing
SV: Structural variant
TSG: Tumor suppressor gene
WGS: Whole genome sequencing
zfpkm: Z-score FPKM
