# Supplementary material for: Systematic discovery of gene fusions in pediatric cancer by integrating RNA-seq and WGS
Source: BMC Cancer. 2023 Jul 3;23:618. doi: 10.1186/s12885-023-11054-3 (PMC10318758; doi:10.1186/s12885-023-11054-3)
Supplement: Supplementary file 1 — Additional file 1: Supplementary material. Supplementary Methods. Supplementary Results. Figure S1. Gene fusions supported by one or more SV tools. Figure S2. Allele fraction of distinct fusions per SV type. Figure S3. Filtering predicted gene fusions to a high confidence subset. Figure S4. High gene fusion burden is associated with copy number instability. Figure S5. Osteosarcomas with TP53 gene fusion. Figure S6. Potentially pathogenic gene fusion candidates in individual patients. Figure S7. HOXA9 gene expression and gene fusion status. Figure S8. Association between ZBTB20 gene expression and survival. Figure S9. Co-occurring fusions and SNVs indicative of TSG disruption. Figure S10. MTAP--CDKN2B-AS1 fusions and associated expression changes of CDKNB-AS1 and CDKN2A. References for supplementary methods and results. [file 12885_2023_11054_MOESM1_ESM.pdf]

Supplementary materials

Table of Contents

Supplementary Methods 1

Supplementary Results 12

Supplementary Figures 17

Figure S1: Gene fusions supported by one or more SV tools. 17

Figure S2: Allele fraction of distinct fusions per SV type. 18

Figure S3: Filtering predicted gene fusions to a high confidence subset. 19

Figure S4: High gene fusion burden is associated with copy number instability. 20

Figure S5: Osteosarcomas with *TP53* gene fusion. 21

Figure S6: Potentially pathogenic gene fusion candidates in individual patients. 22

Figure S7: *HOXA9* gene expression and gene fusion status. 23

Figure S8: Association between *ZBTB20* gene expression and survival. 24

Figure S9: Co-occurring fusions and SNVs indicative of TSG disruption. 25

Figure S10: *MTAP--CDKN2B-AS1* fusions and associated expression changes of *CDKNB-AS1* and *CDKN2A*. 26

References 27

Supplementary Methods

Sample preparation and sequencing

Data was collected as part of the biobanking initiative at the Princes Máxima Center for Pediatric Oncology, and resulted in a pan-cancer cohort of 128 patients. The inclusion criteria used were: the availability of informed consent, paired tumor-normal sequencing WGS data and RNA-seq data of the tumor of sufficient quality (see quality control metrics), and the sample being representative of the cancer type group (i.e. presence of tumor material in the sample).

Following the institute's standardized biobanking protocols [1], RNA and DNA were isolated from fresh frozen tumor tissue and as a matching normal, DNA was isolated from whole blood. Blood and bone marrow samples were enriched for monocytic cells using Ficoll. Total RNA was isolated from tumor samples using the AllPrep DNA/RNA/Protein Mini Kit (QIAGEN) according to standard protocol on the QiaCube (Qiagen) RNA-sequencing (RNA-seq) libraries were generated from 300 ng RNA using the KAPA RNA HyperPrep Kit with RiboErase (Roche) and sequenced with NovaSeq 6000 (2x150 bp) (Illumina). DNA was isolated from paired tumor-normal samples also using the AllPrep DNA/RNA/Protein Mini kit. Whole-genome sequencing (WGS) libraries were generated from 150 ng DNA using the KAPA DNA HyperPlus kit and NovaSeq 6000 sequencing platform (Illumina).

RNA and WGS sequencing data pre-processing

Pre-processing of RNA-seq and WGS was done with the institute's standardized pipelines implementing GATK 4.0 best practices for variant calling using a wdl and cromwell-based workflow [2, 3]. Data quality was assessed with Fastqc (version 0.11.5) to calculate the number of sequencing reads [12]. Picard (version 2.20.1) for both WGS and RNA metrics output such as insert size and MarkDuplicates [13]. The RNA sequencing reads were aligned using Star (version 2.7.2b) to GRCh38 and gencode version 31 [14]. WGS reads

were aligned using BWA mem (0.7.13) to GRCh38. Quality control (QC) metrics are available for all samples (Additional file 2). For WGS, a minimum median coverage of 25x for normal samples and 60x for tumor samples was used. The percentage of duplicate reads was reasonable as well with median 8% and maximum 13% for tumor samples, 7% and 13% for normal samples respectively. Patient M129AAA was resequenced once to achieve sufficient coverage. For RNA data, a minimum of 30 million unique reads was used based on the Picard total reads and percentage of duplicates.

Diagnostic process

As part of the institute's routine diagnostic process, patients were diagnosed according to ICD-O-3 guidelines combining histopathological and molecular characteristics by a pathologist and molecular tumor board. To achieve sufficient sample size for some of the downstream analyses, the ICD-O-3 primary cancer type groups were further grouped into three cancer type supergroups: hemato, neuro and solid. Hemato contains the leukemia (1) and lymphoma (2) primary groups, Neuro the CNS tumors (3) and neuroblastomas (4). Note that there were no patients with retinoblastoma (5) in our cohort. The solid group is composed of all other primary cancer groups (6-12).

Variant calling

Gene fusion predictions were obtained from tumor RNA-seq using STAR-Fusion (version 1.8.0) [4] and GRCh38/Gencode v31 CTAT Oct 2019 and FusionCatcher (version 1.33) [5] with Ensembl v97. Note that the initial input dataset of 129 individuals was reduced to 128, as FusionCatcher failed to succeed on sample M533AAA. Fusion predictions involving human leukocyte antigens or mitochondrial genes are filtered out, but no other pre-filtering based on RNA support was done.

Single nucleotide variants (SNVs) were inferred from paired tumor-normal WGS by Mutect2 from GATK 4.1 [6] and pathogenicity was predicted by variant effect predictor (VEP) (version

92)[7] , according to the GATK4 standards. Somatic SNVs were filtered based on tumor variant allele frequency (AF) > 0.05 and predicted impact (MODERATE or HIGH). Somatic copy number alterations (CNAs) were identified with the GATK4 pipeline according to their standards. We generated a panel of normals (PON) from 18 normal samples prepared and sequenced under the same conditions and used this for normalization. The allelic imbalance ratios were calculated using 1000 genomes, autosomal SNP sites with a minor allele frequency (MAF) > 0.1.

Structural variants (SVs) were inferred from paired tumor-normal WGS using Manta [8] (version 1.6), DELLY [9] (version 0.8.1) and GRIDSS [10] (version 2.7.2). Due to technical issues with running the tool, no GRIDSS output was available for four patients (M863AAC, M479AAA, M156AAA, M606AAA). SVs were not filtered based on quality, read support or somatic/germline annotation. Since these tools vary in how they classify SVs as somatic or germline, we performed this classification based on variant allele fraction (AF) of the paired tumor/normal samples.

The calculation of variant AF was done in agreement with the developer's recommendations for every tool. In the case of Manta, the tool outputs separate files with "somatic SVs" for the tumor and "diploid SVs" for the normal sample. The tumor and normal AF was calculated for the variants in the somatic file using the number of spanning read pairs and split reads that strongly (Q30) support the reference or variant alleles. Variant AF = SRV+PRV / (SRR + PRR + SRV + PRV). For the variants scored under the diploid assumption, only normal AF could be calculated.

For DELLY and GRIDSS the AF was calculated for all variants. DELLY recommends reference/variant allele supporting pairs for imprecise variants (AF = DV/(DR+DV)) and reads for precise variants (AF = RV/(RR+RV)). GRIDSS recommends using the supporting fragments (VF) that combine split reads, discordant pairs and assembly-based support. (AF

= VF / (VF + REF + REFPAIR) for variants larger than the max fragment size distribution, and excluding the REFPAIR for smaller variants (<1000bp).

Fusion-sq algorithm

Intersect predictions from STAR-Fusion and FusionCatcher

As an initial filtering step, we intersected gene fusion predictions from STAR-Fusion and FusionCatcher based on the combination of up- and downstream (5'/3') partner genes. To accommodate for algorithmic differences between tools, we matched fusions based on gene pairs and allowed for reciprocal orientations (3'--5' fusions).

Prepare RNA/DNA matching intervals

Gene fusion predictions from RNA-seq were used to derive genomic intervals for SV breakpoint matching taking into account intron-exon gene structure. First, transcripts were retrieved from the ENSEMBL database based on ENSEMBL gene stable identifiers or based on genomic location in case an identifier was lacking (e.g. immunoglobulin genes). Second, a hierarchy of matching intervals was generated, from more to less precise: 1) intron adjacent to the RNA breakpoint, 2) alternative splice junction +/-10 bp from breakpoint, 3) flanking interval spanning +/-500bp at each side of the breakpoint (within the gene body if available), 4) RNA breakpoint to start/end of the gene body. The adjacent intron interval can differ between transcripts, therefore the union of introns was used for initial matching of RNA and SV breakpoints. The transcript-specific intervals later used to match SV breakpoints to individual transcripts.

Match SVs to gene fusions

SVs identified by Manta, DELLY and GRIDSS were matched to gene fusion predictions based on these genomic intervals. Note that respecting the hierarchy of the intervals is important because of the inherent overlap between the intervals, i.e. introns fall inside the

gene body. We conclude that fusions are validated by WGS if SVs are found that link the 5'/3' partner genes by any of these genomic intervals.

If no SV was identified that directly links the 5' and 3' gene, an attempt was made to resolve the fusion by a composite of two SVs. All SV breakpoints originating in respectively the 5' and 3' partner gene's adjacent intron/flanking/splice-junction intervals were considered. Fusions were flagged as 'composite' when two SVs were identified that respectively originate from the 5'/3' partner genes and have their "other end" in close proximity (5kb) therefore indirectly but effectively linking the partner genes to form a fusion.

<u>Combine supporting SVs</u>

As the final step of the pipeline, the supporting SVs from the different tools were integrated for the WGS validated fusions. Each fusion was annotated with the genomic breakpoints and SV characteristics (i.e. SV type, size, tumor and normal allele fractions, breakpoint quality filters). Also, the SV breakpoints were used to select corresponding transcripts for the partner genes and annotated with the involved exons and gene fragments based on this selection. This step also further annotates the precision of SV support by distinguishing between SVs that link the 5'/3' partner genes via introns of individual transcripts *(gup/gdw_location = intron)* and SVs that link via the union of introns used during matching but do not satisfy this strict criterium for both partner genes (*gup/gdw_location = intron_consensus)*. In some cases, multiple fusion predictions were validated by the same SV and subsequently considered as a single fusion. Vice versa, multiple SVs could be linked to a single fusion prediction as well.

We further selected high confidence fusions (hcFs) based on the support by at least two SV tools and the location of the SV breakpoints relative to the chimeric transcript. Fusions were labeled as '*precise_location*' in case the SVs link partner genes by their adjacent introns, flanking regions or alternative splice junctions. To be considered as *'high_confidence'*, these

supporting SVs had to be resolved as the same event by at least two tools based on 50% reciprocal overlap and matching SV type. SV breakpoints smaller than 30bp were resized to 30bp during matching. In case multiple high confidence SVs support the fusion, the SV with the highest tumor AF is selected.

Fusions were classified based on AF after resolving the underlying high confidence SVs, since this filters out potential additional lower confidence SVs. Fusions were classified as tumor-specific, (likely) germline and low AF based on mean tumor and normal AF of the associated SVs.

- Tumor specific: (tumor AF - normal AF) > 0.05 & (tumor-normal)/normal ratio > 1.5
- Germline: normal AF > 0.05 & (tumor-normal)/normal ratio < 1.1
- Low AF: (tumor AF - normal AF)<0.05 & normal AF <0.05

Expression data generation and analysis

Gene expression was analyzed using featureCounts from Rsubread (version 1.32.4) with Gencode v31 CTAT Oct 2019 annotation and settings *allowMultiOverlap=T, largestOverlap=T* and *countMultiMappingReads=F.*

Gene expression alterations were assessed with z-scores of log2 transformed gene length normalized read counts (Fragments Per Kilobase of transcript per Million mapped reads, FPKM). Expression values were first log transformed, after which the group mean and standard deviation were calculated. z-*score = (fpkm - fpkm_mean)/ fpkm_sd.* Gene expression z-scores (zfpkm) were reported relative to the full cohort, cancer type supergroup and the primary cancer group. Normality of the log-transformed FPKMs was assessed with Shapiro for each group separately to assess the validity of using gene expression z-scores for outlier analysis. As threshold for aberrantly expressed genes, +/-1.96 z-score was used corresponding to 95% confidence interval (p<0.05). To account for cases where gene expression is not normally distributed in a certain group, we also assessed whether a patient

carrying a gene fusion has a significantly different gene expression than other patients in their subgroup using Wilcoxon rank sum test.

To study whether fusions with oncogenes or tumor-suppressor genes as partner genes are associated with gene expression changes, we assessed subset enrichments with Fisher's exact test and compared zfpkm distribution amongst subsets of fusions with Wilcoxon rank sum tests.

Overexpression is defined as >1.96 zfpkm relative to the cancer type supergroup. Distributions of zfpkm supergroup scores were compared relative to all tumor-specific high confidence gene fusions (hcTSFs) for fusions with and without downstream oncogenes. Similar analysis was conducted for downregulation of tumor-suppressor genes. Next, we investigated whether expression changes and copy number (CN) changes were associated. Hereto again the Fisher's exact test was used for subset enrichments relative to all hcTSFs and Wilcoxon rank sum tests for changes in zfpkm distributions. CN gain was defined as >0.58 read depth log2 fold change which corresponds to 1.5x fold change, and amplification as >1.58 corresponding to 3x fold change.

SV type and size analysis

SV properties were analyzed for the high confidence gene fusion that have underlying SVs supported by at least two SV tools. To account for differences between how these tools report events, SV properties were harmonized between tools prior to analysis. SV breakpoints were classified into the major types of simple SVs based on their relative orientation: deletions, duplications, inversions and inter-chromosomal translocations. Sizes of SVs were regarded as positive numbers and only considered for intra-chromosomal events. One gene fusion *(AC063944.1--LINC00882*) was resolved as tumor-specific in one patient and as germline in another, therefore it was labeled as ambiguous and ignored during this analysis.

Recurrence analysis

The number of unique occurrences of fusions across patients was used during recurrence analysis. Every occurrence of an upstream-downstream (5'/3') partner gene pair in a patient is counted once, ignoring multiple predicted breakpoints in a single patient. Fusion directionality was respected, so canonical and reciprocal gene fusions were regarded as two distinct events.

Integration of CN data and SVs

Copy number (CN) segments were mapped to SVs based on genomic location. To analyze read depth of SVs, we considered the CN ratio log2 fold change (l2fc) of SV breakpoints and calculated a weighted average from overlapping segments.

Measures of copy number instability

Fraction of genome altered (FGA) was calculated relative to the expected autosomal genome size, excluding alternate loci: 2875001522 bp.

FGA = number of base pairs >0.2 absolute CN l2fc / expected autosomal genome size.

In addition to the FGA, the maximum CN l2fc was used to gauge whether patients had focal amplifications.

The subset "patients with a high gene fusion burden" is defined by the 95th percentile (5 or more) of high confidence gene fusions classified as either tumor-specific or low AF. Patients in this subset: M809AAA, M479AAA, M691AAA, M002AAB, M787AAA, M040AAA, M606AAC, M597AAC.

Annotation of SVs

To verify how SVs link together the fusion partner genes independently of RNA-seq evidence, SVs were annotated with introns based on overlap with canonical transcripts. For each partner gene, a canonical transcript was selected based on stepwise filtering until a single transcript remained: MANE select, the tags "basic, CCDS, APRIS", protein coding, transcript support level and coding sequence length. The transcript annotation was retrieved from Gencode v31 [14].

As an additional confirmation of the classification between tumor-specific and germline SVs based on AF, SVs underlying gene fusions were compared to SVs occurring in the general population. Hereto, SVs were retrieved from NCBI Curated Common Structural Variants (nstd186) [11], gnomAD Structural Variants (nstd166) [12] from NCBI repository and from DGV [13] (version 2020-02-25) accessed on 2021-03-11. SVs supporting gene fusions were matched to population SVs based on 50% reciprocal overlap, regardless of SV types as variant type annotation differs per database and SV detection method. Fusions were flagged in case their underlying SVs matched to population SVs from any of these databases (*anno_sv_population*) (corresponding column of Table 1).

Furthermore, SVs were annotated with repeats and segmental duplications to assess whether their breakpoints reside in rearrangement-prone genomic regions. Hereto, repeats and segmental duplications tracks were retrieved from UCSC table browser accessed on 2021-04-20 [14]. Repeats from RepeatMasker were pre-filtered by repeat class (LINE, SINE, LTR) and completeness (<50 bp of repeats left) to prevent spurious annotations. Gene fusions were annotated with identifiers of repeats and segmental duplications overlapping the underlying SV start/end coordinates (*repeat_family, segdup)*.

Annotation of gene fusions

To identify whether gene fusions were previously reported in either healthy tissue or cancer samples, we compared our findings to chimeric transcript databases. Fusions were

annotated as healthy chimera based on the default annotation from STAR-Fusion [4]. For the annotation of cancer chimera, we used ChimerDB 4.0 (retrieved on 2021-02-17) [15] and the Mitelman database (v20201015, retrieved on 2021-01-07) [16] matching exact gene pairs. *(anno_cancer_chimera).*

To aid the interpretation of gene fusions and select potentially pathogenic gene fusions, we assigned gene-level properties to the 5'/3' partner genes of fusions based on their stable ENSEMBL identifiers and/or gene names. Cancer-related gene datasets were retrieved from COSMIC [17] (cancer gene census v92), OncoKB (accessed on 2021-04-14) [18] and Grobner [19]. For COSMIC and OncoKB we adopted their annotation of oncogenes and tumor-suppressor genes (TSGs). As a pediatric cancer resource, we retrieved recurrently mutated genes identified by Grobner *et al.* and used "amplification" as proxy for oncogene and "deletion/gene-disrupting structural variant" for TSG. Similarly, genes and fusions are annotated as kinase based on the human kinome [20] (retrieved from www.kinase.com on 2021-01-16). For each annotation, it was specified whether the fusion partner gene has that property (*gup_label, gdw_label)*, and the annotation was summarized on the level of the gene fusion for easier selection (*anno_has_onco_or_tsg*, *anno_has_kinase)*. Finally, gene fusions were also annotated with cytobands retrieved from the UCSC table browser on 2021-05-06.

To summarize annotations for visualization and reporting (including in Figure 3 and Tables 1 and 2), we annotated fusions *(annotation)* based on known clinical relevance (clinical), involving a cancer-related gene or cancer chimera (cancer), population SV or healthy chimera (common), or both cancer and common (both). For further analysis, we selected potentially pathogenic fusions based on whether they contain an oncogene or TSG.

Correlating gene expression and prognosis

Kaplan Meier plots were retrieved from the R2 genomics analysis and visualization platform.

(http://r2platform.com/, accessed on 2021-07-12). Results were obtained with KaplanScan, which calculates the optimum gene expression threshold for survival analysis with statistical testing. Data from neuroblastoma samples in the publicly available Versteeg dataset was used for this analysis http://www.ncbi.nlm.nih.gov/geo/query/acc.cgi?acc=GSE16476

Gene fusion schematics

Gene fusion schematics were generated with Protein Paint from the St. Jude cloud (https://proteinpaint.stjude.org/ [21, 22] using the default RefSeq transcripts in hg38 and genomic coordinates of the underlying SVs resolved with Fusion-sq.

Supplementary Results

In addition to the identification of known clinically relevant gene fusions we identified several high confidence tumor specific gene fusions which are likely to be damaging based on the disease mechanisms that either activate a known oncogene or kinase gene, or disrupt a known tumor suppressor gene.

Activation of oncogenes

Overexpression of oncogenes resulting from gene fusions is often suggested as an activation mechanism[23]. In a subset of patients, we resolved fusions that potentially activate 3' oncogenes as reflected in expression changes or result in a gain of function of a transcription factor (Fig. 5).

M911AAA: The *PAX3--WWTR1* fusion was found in an embryonal rhabdomyosarcoma patient (M911AAA) resulting from a translocation t(2;3) (tumor AF 0.81) which gives rise to a fusion product involving exons 1-7 of *PAX3*. Canonical *PAX3--FOXO1* fusions involve the same *PAX3* exons, which suggests that *PAX3--WWTR1* may have similar functional effects as the *PAX3/7* driver gene fusions in alveolar rhabdomyosarcoma (Additional file: Figure S6a).

M385AAA: In a patient with pre-T-cell lymphoblastic leukemia (M385AAA), we identified *MED14--HOXA9* as in-frame gene fusion involving the homeobox transcription factor domain (tumor AF 0.47, normal AF 0.06, Additional file 1: Figure S6bd) and associated overexpression of *HOXA9* (2.2 zfpkm, p=0.15). In this case, *HOXA9* overexpression did not reach significance because an acute myeloid leukemia patient (M975AAA) with a *NUP98--NSD1* fusion, which is known to dysregulate *HOXA* genes, also has a high *HOXA9* expression (3.0 zfpkm, Additional file 1: Figure S7)[24]. While this specific fusion has not yet been reported, diverse mechanisms of *HOXA9* activation might impact treatment and prognosis in T-cell leukemia[25, 26]. Notably, a *FLT3-ITD* was identified in this patient too,

and concurrent *FLT3-ITD* and *HOXA9* overexpression was suggested to be potentially pathogenic in acute myeloid leukemia[27].

M637AAB: We identified a fusion associated with *TERT* overexpression in a patient diagnosed with liver cell adenoma (M637AAB, Additional file 1: Figure S6c). This fusion warrants further investigation, because *TERT* activation may be a factor contributing to malignant transformation to liver carcinoma[28].

M014AAA: In a yolk sac tumor (M014AAA), we identified a gene fusion with the *ERBB4* kinase, which was previously found to be overexpressed in yolk sac germ cell tumors[29] and suggested as a potential drug target[30]. Although the kinase domain is not involved in this patient's gene fusion (Additional file: Figure S6d), also kinase-dead mutants can have functional consequences in cancer[31].

Activation of kinase genes

Gene fusions can also be pathogenic through activation of kinases due to e.g. loss of an auto-inhibitory domain or increased dimerization. Of the eight hcTSPs involving kinases two fusions resulted in the chimera containing the kinase domain *MEF2A--IGF1R* and *TNK1--GPS2* (Additional file: Figure S6e-f).

M999AAA: The *MEF2A--IGF1R* was resolved in an Embryonal rhabdomyosarcoma in an amplified region (CN l2fc 4.8) with high expression of both partner genes (2.5 and 4.4 zfpkm respectively, $p<0.1$). Although the underlying 707 kb DUP has a very low AF of 0.006, this is consistent with the amplification. The breakpoints indicate the UTR of *MEF2A* is fused to IGF1R c-term with its kinase domain, and the resulting chimera seems to be actively transcribed (8 FFPM). Both the chimeric protein and overexpression suggest a possible IGF1R kinase activation event and that the patient might benefit from targeted therapy [32].

M952AAA: The fusion *TNK1--GPS2* was found in a patient with anaplastic large cell lymphoma resulting from a 74 kb inversion (0.40 tumor AF). Resolving the underlying SV allowed us to confirm the presence of this gene fusion which was predicted with different breakpoints and in reciprocal orientations by the RNA tools (STAR-Fusion predicted *GPS2--TNK1* and FusionCatcher *TNK1--GPS2*). This fusion and other rearrangements involving these genes have been reported in this cancer type [33] and were suggested to either disable both genes (which can act as tumor suppressors) but also give rise to a C-term truncated form of *TNK1* with oncogenic properties. Whilst the SV breakpoint inside GPS2 might disrupt its function, it is clear that the kinase *TNK1* is over expressed (2.3 zfpkm $p<0.1$) and C-term truncated.

Fusions disrupting tumor-suppressor genes

Gene fusions that involve tumor-suppressor genes (TSGs) are potentially pathogenic, as the underlying SVs can disrupt the TSGs. Similar to oncogenes, we did not find a general trend of downregulation in the 19 fusions involving TSGs, however SVs intersecting a gene can be disruptive regardless of whether gene expression is affected. Three specific cases did show a significant decrease in gene expression ($p<0.1$) relative to the cancer type supergroup: ***ZBTB20***--*LSAMP* (-4.0 zfpkm, $p<0.05$), ***NF1***--*RAB11FIP4* (-2.4 zfpkm) and the previously mentioned ***ATRX***--*LINC01280* (-3.5 zfpkm) in a patient with osteosarcoma. Both *ZBTB20--LSAMP* and *NF1--RAB11FIP4* have been previously detected in other adult cancer types[15, 16]. In addition, *ZBTB20* and *NF1* play important roles as TSG in pediatric cancers and *LSAMP* is suggested as a potential TSG in osteosarcoma and neuroblastoma[34–36]. However, they have not yet been described as markers of TSG disruption in pediatric cancers.

M909AAA: In a neuroblastoma patient (M909AAA), we resolved a *ZBTB20--LSAMP* fusion due to a 1 Mb duplication which indicates a potentially pathogenic disruptive event (Additional file 1: Figure S6g). *ZBTB20* is associated with neuronal differentiation and disruption of this process is a known oncogenic factor in neuroblastoma[37]. The neural cell-adhesion protein *LSAMP* has been suggested as a potential TSG in neuroblastoma[34, 36] as well as other cancer types such as osteosarcoma[35], however the mechanism is less clear. Both *ZBTB20* and *LSAMP* are downregulated and for *ZBTB20* low expression is correlated with poor prognosis in a publicly available neuroblastoma dataset (Additional file 1: Figure S8)[37].

M535AAA: The *NF1--RAB11FIP4* fusion identified in a patient with Pleomorphic xanthoastrocytoma (M535AAA), was caused by a 185 kb deletion with a disruptive effect on *NF1* (Additional file 1: Figure S6h). *NF1--RAB11FIP4* fusions have been previously detected in multiple cancer types[15]. *NF1* is also an important TSG that is recurrently mutated in pediatric CNS tumors[38] and in rare instances also specifically xanthoastrocytoma[39]. Of note, germline *NF1* alterations in combination with a second-hit somatic mutation can indicate sensitivity to immunotherapy[38]. Although we only identified a somatic deletion with 0.5 tumor AF, the expression analysis provides additional evidence that the *NF1* gene is significantly disrupted in this patient.

To investigate whether TSGs could be disrupted by gene fusions without this being reflected in gene expression changes, we sought to identify co-occurring somatic single nucleotide variants (SNVs). We found co-occurring fusions and SNVs for *RB1* and *NCOR1* that could indicate double-hits of these TSGs, despite the fact that no reduction in gene expression was observed for *RB1* (-0.1 zfpkm) and only a minor reduction for *NCOR1* (-1.2 zfpkm).

M152AAD: In an adrenal cortical carcinoma patient (M152AAD), the *RB1* gene was affected by a translocation resulting in the *RB1--DGKB* fusion and by a splice donor site mutation (chr13:48473390_GGTGA/G, tumor AF 0.32, normal AF 0, Additional file 1: Figure S9a).

M930AAB: In a second patient with pre B-cell lymphoblastic leukemia (M930AAB), we identified a 158 kb deletion in the *NCOR1* locus resulting in the fusion *NCOR1--ZWIM7,* as well as a frameshift mutation (chr17:16070282_C/CT, tumor AF 0.85, normal AF 0.03, Additional file 1: Figure S9b). This deletion overlaps a healthy population SV and is likely ALU-mediated given that the breakpoints fall in ALU repeats. However, in this case, the deletion is specific to the patient's tumor sample (0.33 tumor AF, 0 normal AF). Also, for both *RB1* and *NCOR1*, gene fusions with different 3' partner genes have been previously identified in cancer[15, 16]. In combination, the co-occurrence of deleterious SNVs and the promiscuity of these fusions seem to suggest that the presence of these gene fusions is indicative of TSG disruption.

M266AAA & M345AAA: Finally, TSG disruption can also occur through alternative mechanisms as with the *MTAP--CDKN2B-AS1* fusions. These were the only recurrent fusions in our cohort and have been previously reported in melanoma[40]. The presence of a *MTAP--CDKN2B-AS1* fusion indicates disruption of the *CDKN2A* locus via two possibly parallel mechanisms; directly resulting from the deletions in the *CDKN2A* locus causing the gene fusion, and indirectly through upregulation of *CDKN2B-AS1* (0.9-2.0 zfpkm, Additional file 1: Figure S10) which can have a repressive effect via Polycomb or RNA interference[40, 41]. Therefore, presence of *MTAP--CDKN2B-AS1* fusions can indicate concurrent *CDKN2A* disruption on multiple regulation levels. Taken together, these examples illustrate that resolving underlying SVs can provide crucial orthogonal support and additional evidence for TSG disruption, thereby facilitating mechanistic understanding and clinical interpretation.

Supplementary Figures

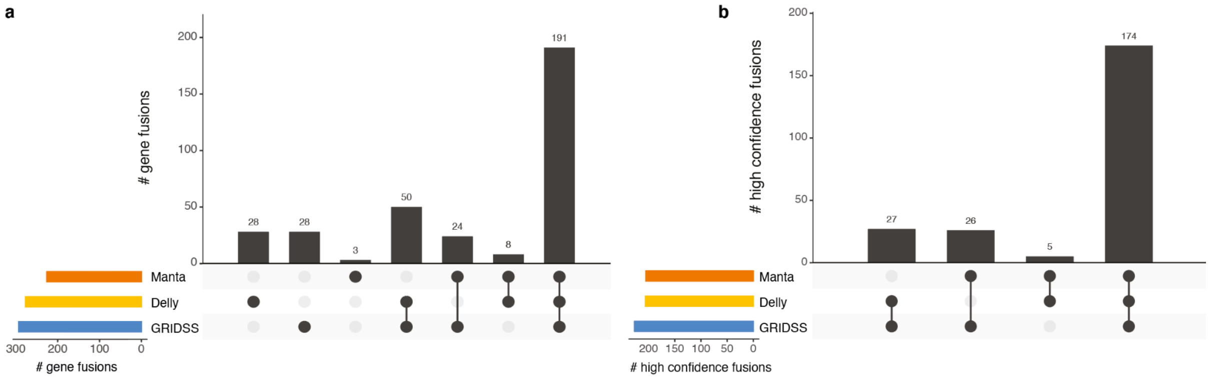

Figure S1: Gene fusions supported by one or more SV tools.

a Distribution of gene fusions supported by SVs detected by Manta, DELLY and/or GRIDSS.

b same as **a** but for the high confidence subset.

NB: no GRIDSS output was available for four patients (M863AAC, M479AAA, M156AAA, M606AAA)

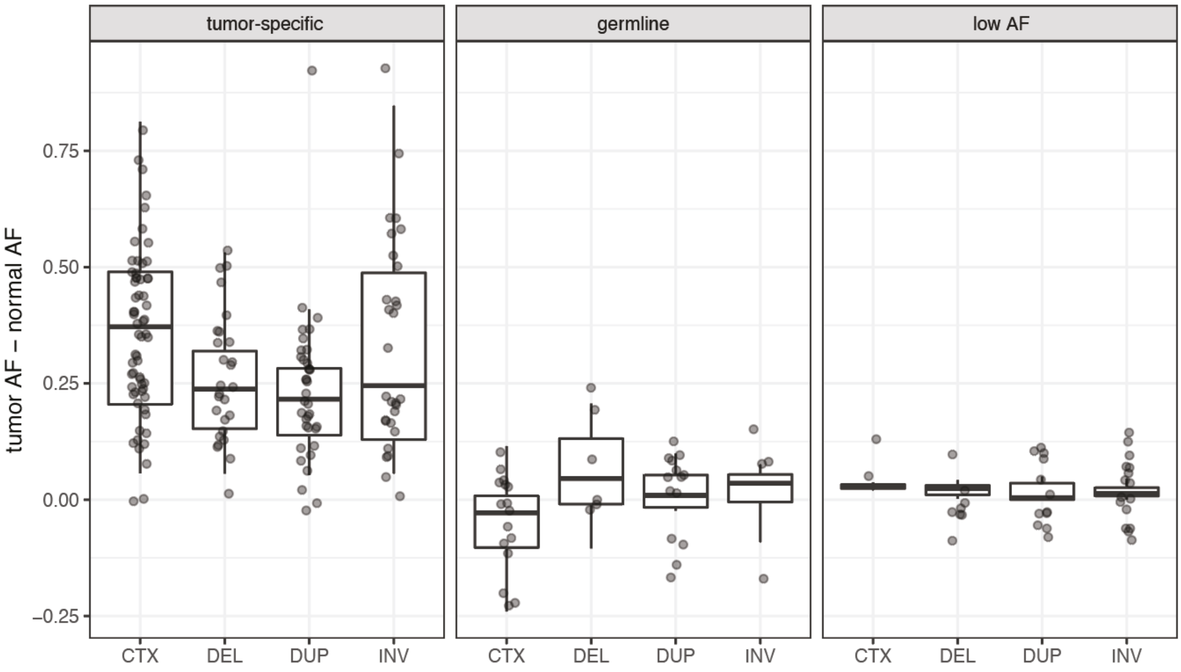

Figure S2: Allele fraction of distinct fusions per SV type.

Allele fractions (AF) of distinct fusions are shown for either tumor-specific (left), likely germline (middle) or low AF (right).

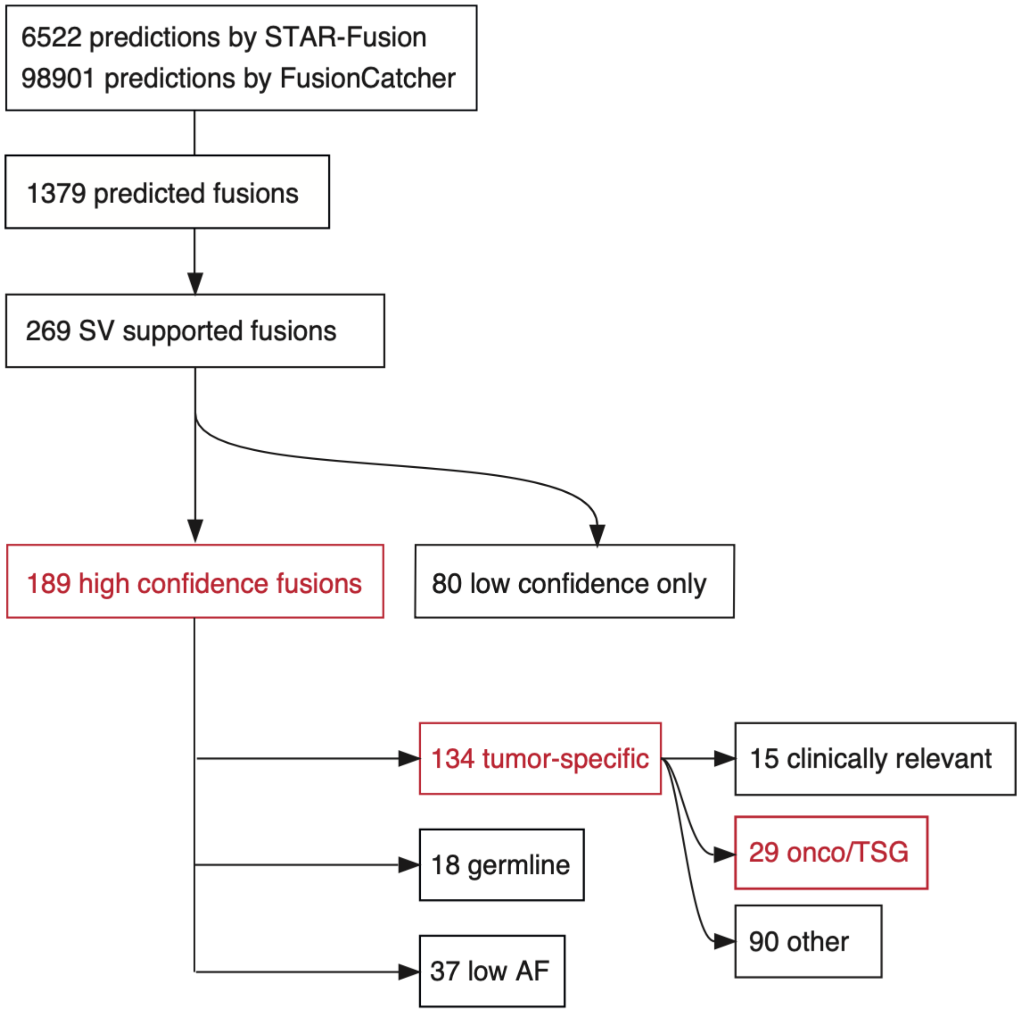

Figure S3: Filtering predicted gene fusions to a high confidence subset.

Schematic overview of the number of distinct fusions throughout the Fusion-sq pipeline. The steps are the same as in Fig. 1b, but gene fusions are mapped to “distinct fusions” such that fusions occurring in multiple patients are merged and counted only once. Subsets discussed in the main text are highlighted in red and available in Table 3.

Note that the 27 distinct fusions involving an oncogene or tumor-suppressor gene (onco/TSG) in patients in the main text specifically refers to those identified in patients without known clinically relevant gene fusion. The other 2 out of 29 fusions are detected in patients carrying a known clinically relevant gene fusion and therefore not discussed in the main text.

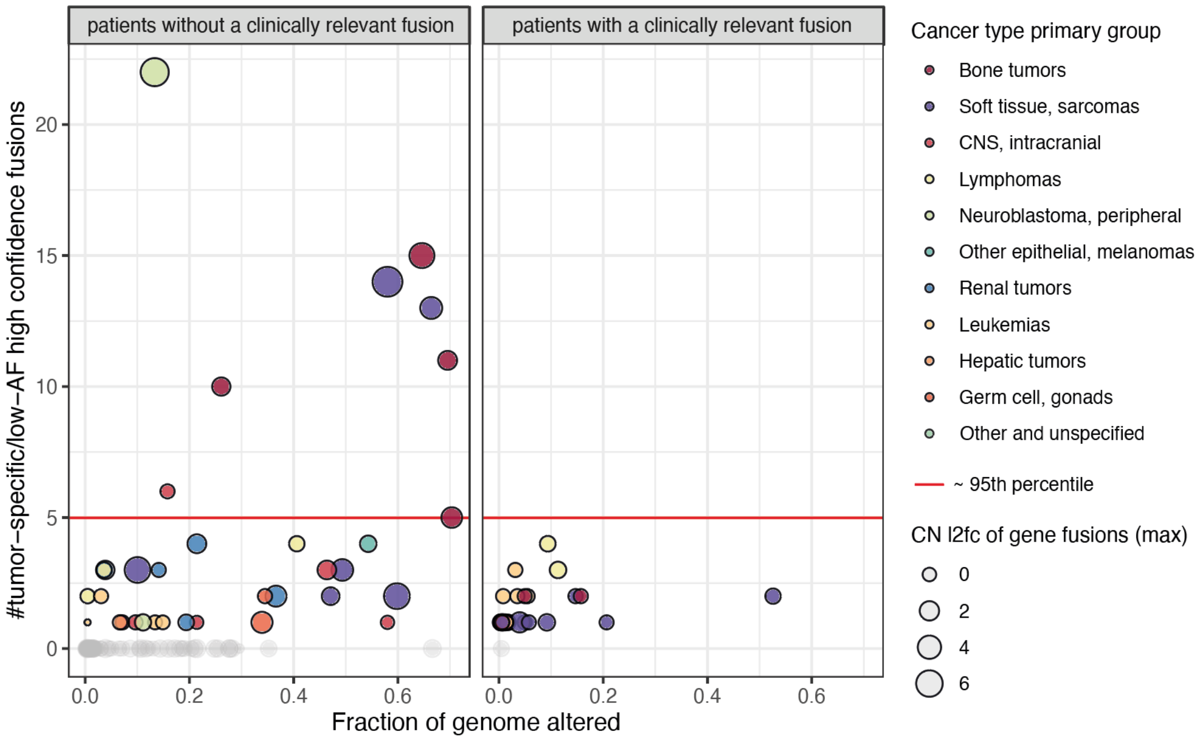

Figure S4: High gene fusion burden is associated with copy number instability.

Relationship between the fraction of genome altered by copy number alterations (FGA) and the number of high confidence gene fusions classified as tumor-specific or low AF. Colors represent primary cancer type groups, circle size corresponds to the maximum copy number log2 fold change (CN l2fc) underlying the gene fusion. Red line indicates >=5 corresponding to the 95th percentile. Patients equal or higher are labeled as high gene fusion burden (Methods) and have either a high FGA or gene fusions arising from focal amplifications (high CN l2fc).

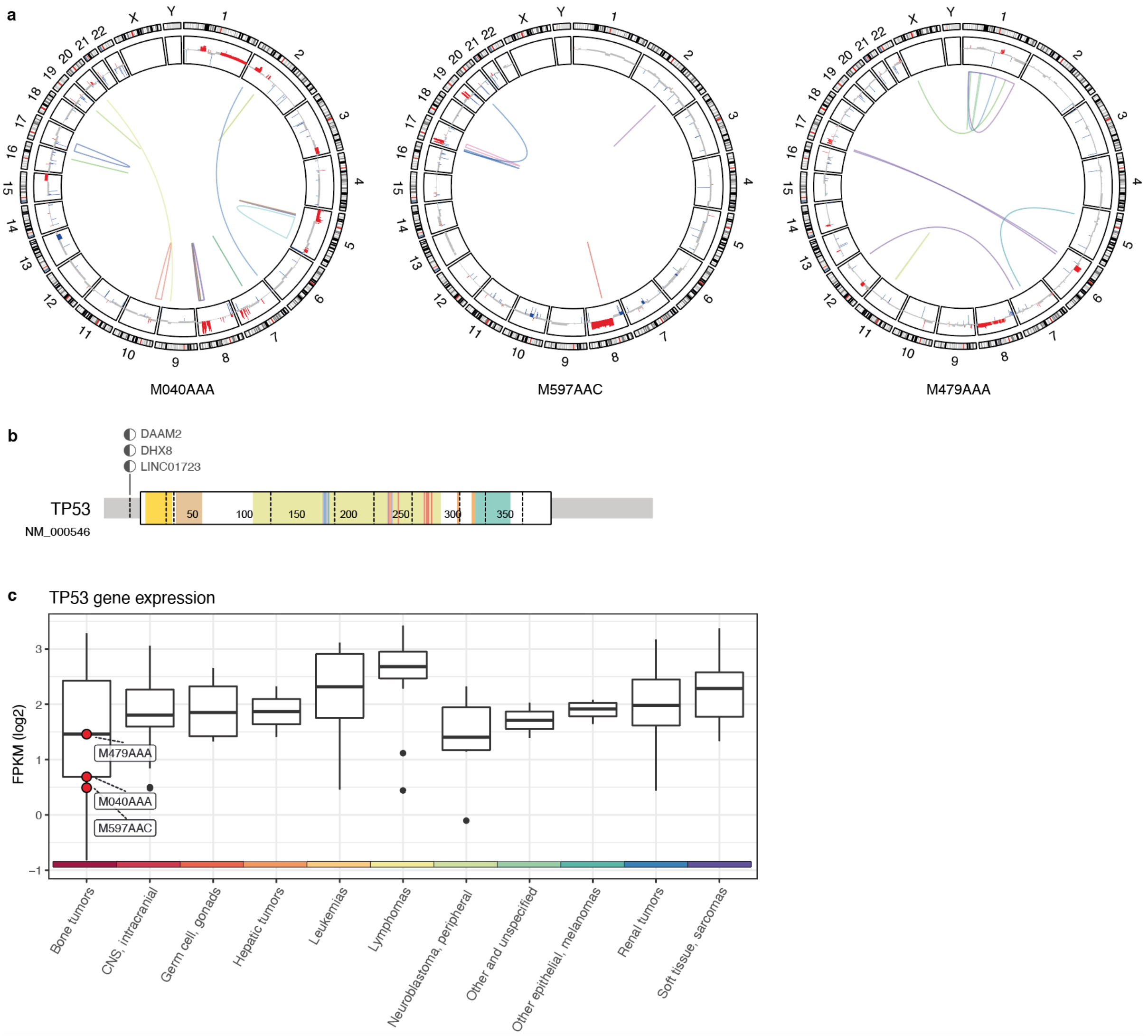

Figure S5: Osteosarcomas with *TP53* gene fusion.

a Circos plots for patients carrying a *TP53* gene fusion (M479AAA, M597AAC, M040AAA) with high confidence tumor-specific gene fusions (multi-colored links), copy number gains (red) and losses (blue). **b** Schematic representation of the resolved gene fusions with *TP53* exon 1 and downstream (3') partner genes. **c** Gene expression levels of *TP53* (log2 FPKM) of patients carrying a *TP53* gene fusion (red circles) in comparison to all patients split according to their primary cancer type group (box plots). Colored bar at the x-axis indicates the primary cancer type group.

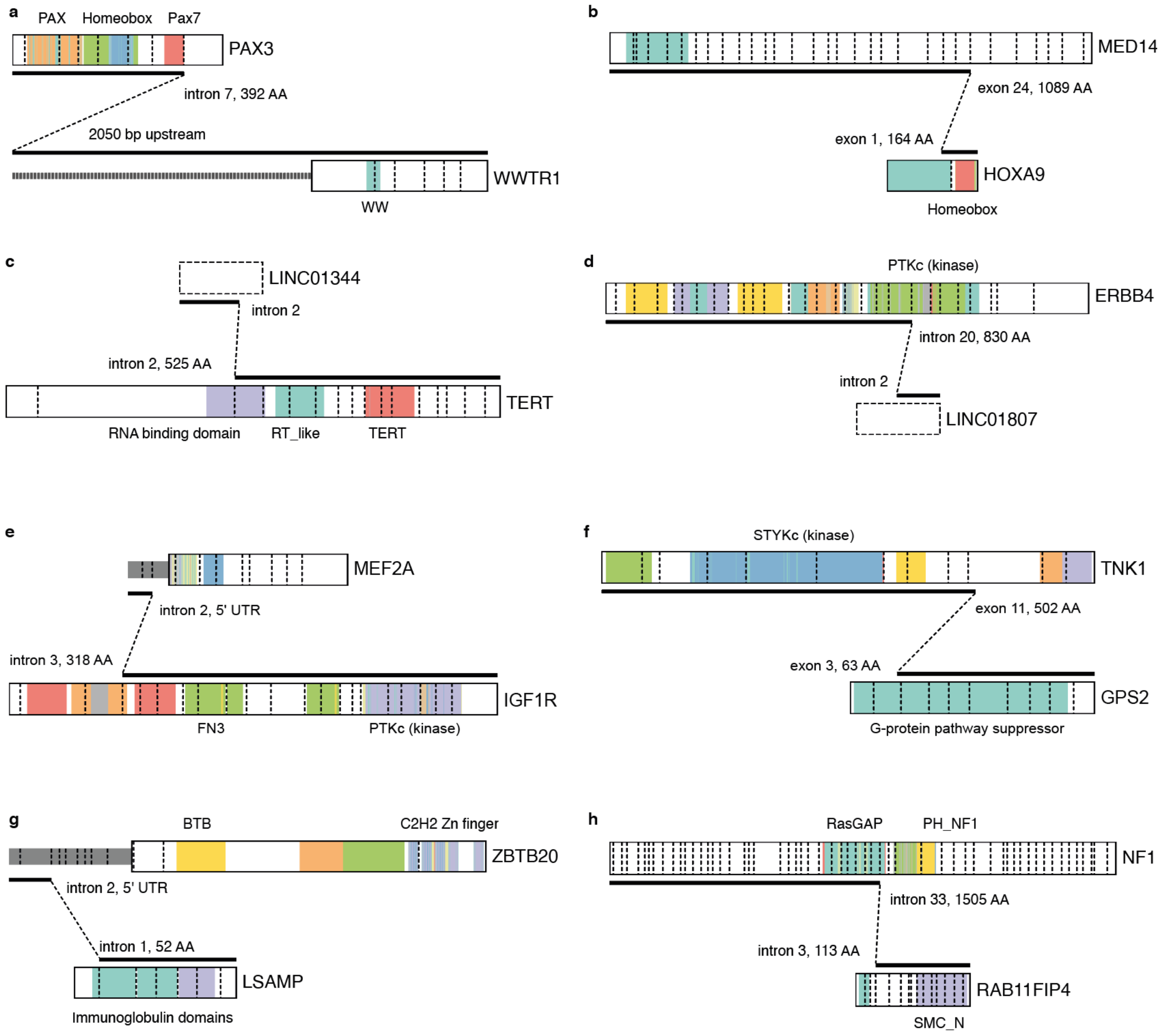

Figure S6: Potentially pathogenic gene fusion candidates in individual patients.

Schematic representations of gene fusions: *PAX3--WWTR1* (**a**), *MED14--HOXA9* (**b**), *LINC01344--TERT* (**c**), *ERBB4--LINC01807* (**d**), *MEF2A--IGF1R* (**e**), *TNK1--GPS2* (**f**), *ZBTB20--LSAMP* (**g**) and *NF1--RAB11FIP4* (**h**).

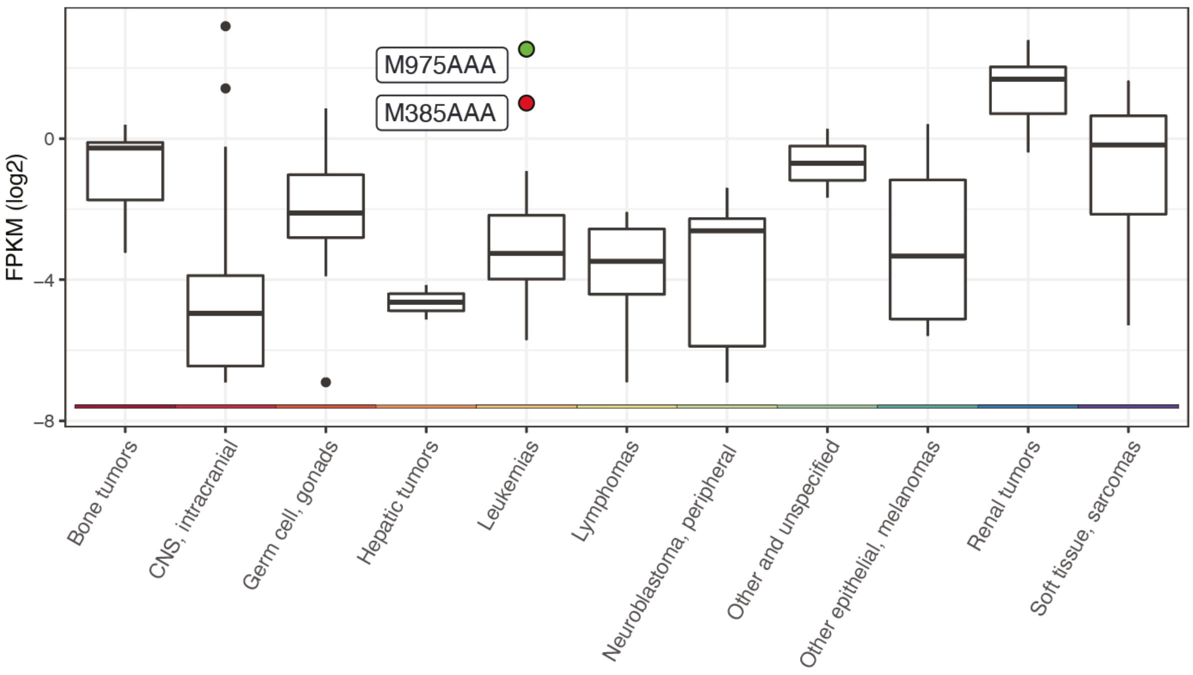

Figure S7: *HOXA9* gene expression and gene fusion status.

Gene expression levels of *HOXA9* (log2 FPKM) for patient M385AAA carrying a *MED14--HOXA9* gene fusion (red circle), patient M975AAA with a *NUP98--NSD1* gene fusion (green circle) and all patients split according to their primary cancer type group (box plots).

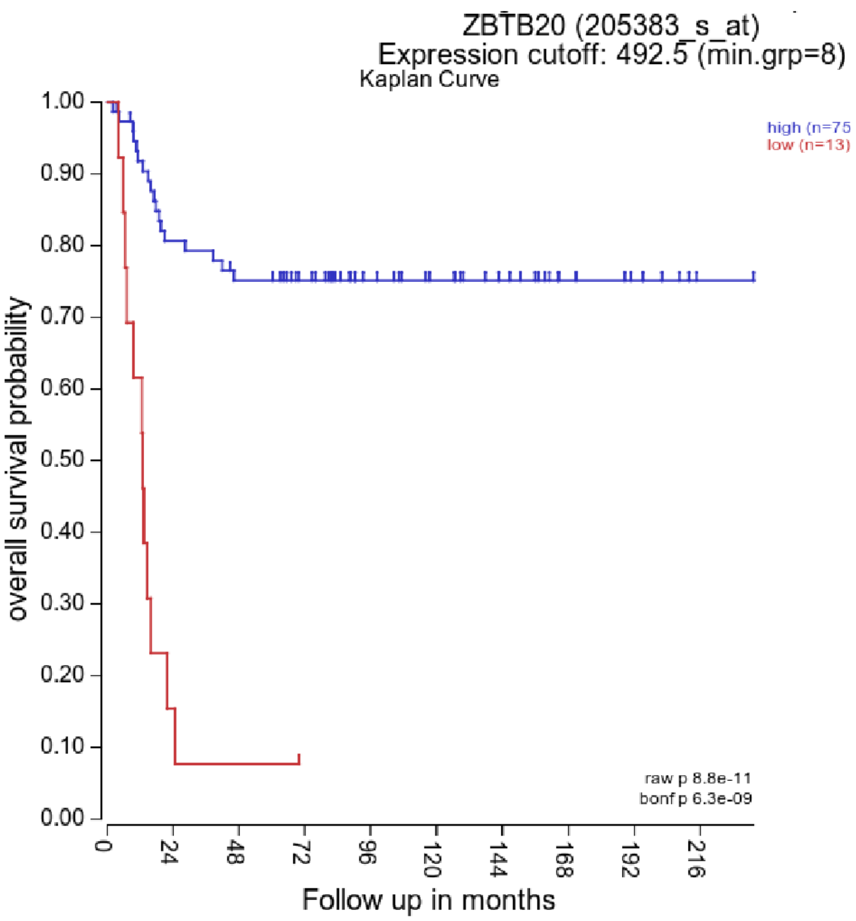

Figure S8: Association between *ZBTB20* gene expression and survival.

Kaplan-Meier curves with overall survival data of neuroblastoma samples from a publicly available dataset [37] separated into high and low expression of ZBTB20 based on the threshold obtained with KaplanScan. Data and plots are retrieved from the R2 platform [42].

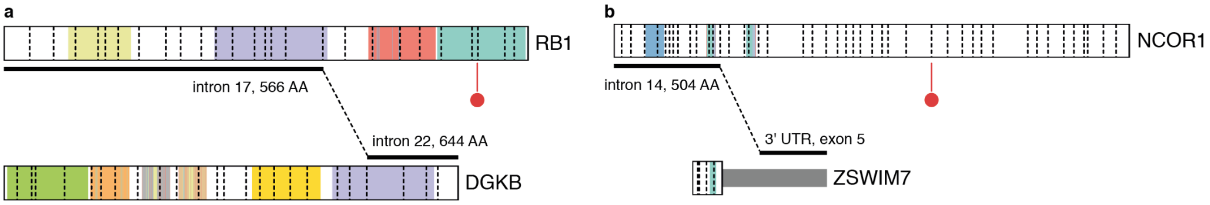

Figure S9: Co-occurring fusions and SNVs indicative of TSG disruption.

Schematic representation of gene fusions and somatic SNVs (red flags) detected in the same patient for **a** *RB1--DGKB* in patient M152AAD and a splice donor site mutation (chr13:48473390_GGTGA/G) and for **b** *NCOR1--ZSWIM7* in patient M930AAB and a frameshift mutation (chr17:16070282_C/CT)

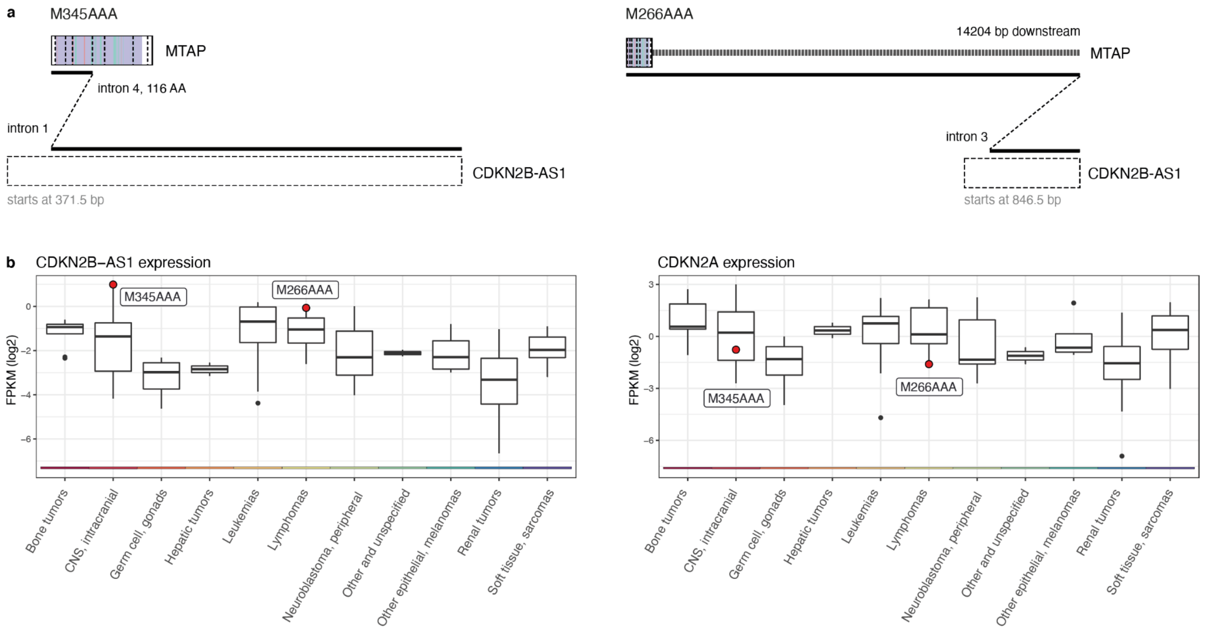

Figure S10: *MTAP--CDKN2B-AS1* fusions and associated expression changes of *CDKNB-AS1* and *CDKN2A.*

a Fusion schematics of *MTAP--CDKN2B-AS1* fusions generated using ProteinPaint from St. Jude Cloud [21, 22] from patients M345AAA (left) and M66AAA (right). **b** Gene expression levels (log2 FPKM) of *CDKNB-AS1* (left) and *CDKN2A* (right) for patient M345AAA and M66AAA carrying a *MTAP--CDKN2B-AS1* gene fusion (red circles) and all patients split according to their primary cancer type group (box plots).

References

1. Hehir-Kwa JY, Koudijs MJ, Verwiel ETP, Kester LA, van Tuil M, Strengman E, et al. Improved Gene Fusion Detection in Childhood Cancer Diagnostics Using RNA Sequencing. JCO Precis Oncol. 2022;6:e2000504.

2. Van der Auwera GA, Carneiro MO, Hartl C, Poplin R, Del Angel G, Levy-Moonshine A, et al. From FastQ data to high confidence variant calls: the Genome Analysis Toolkit best practices pipeline. Curr Protoc Bioinformatics. 2013;43:11.10.1–11.10.33.

3. Kerstens HHD, Hehir-Kwa JY, van de Geer E, van Run C, Verwiel ETP, van der Leest D, et al. Trecode: a FAIR eco-system for the analysis and archiving of omics data in a combined diagnostic and research setting. bioRxiv. 2020;:2020.11.13.363689.

4. Haas BJ, Dobin A, Stransky N, Li B, Yang X, Tickle T, et al. STAR-Fusion: Fast and Accurate Fusion Transcript Detection from RNA-Seq. bioRxiv. 2017;:120295.

5. Nicorici D, Şatalan M, Edgren H, Kangaspeska S, Murumägi A, Kallioniemi O, et al. FusionCatcher – a tool for finding somatic fusion genes in paired-end RNA-sequencing data. bioRxiv. 2014;:011650.

6. Benjamin D, Sato T, Cibulskis K, Getz G, Stewart C, Lichtenstein L. Calling Somatic SNVs and Indels with Mutect2. bioRxiv. 2019;:861054.

7. McLaren W, Gil L, Hunt SE, Riat HS, Ritchie GRS, Thormann A, et al. The Ensembl Variant Effect Predictor. Genome Biol. 2016;17:122.

8. Chen X, Schulz-Trieglaff O, Shaw R, Barnes B, Schlesinger F, Källberg M, et al. Manta: rapid detection of structural variants and indels for germline and cancer sequencing applications. Bioinformatics. 2016;32:1220–2.

9. Rausch T, Zichner T, Schlattl A, Stutz AM, Benes V, Korbel JO. DELLY: structural variant

discovery by integrated paired-end and split-read analysis. Bioinformatics. 2012;28:i333–9.

10. Cameron DL, Schröder J, Penington JS, Do H, Molania R, Dobrovic A, et al. GRIDSS: sensitive and specific genomic rearrangement detection using positional de Bruijn graph assembly. Genome Research. 2017;27:2050–60.

11. dbVar. https://www.ncbi.nlm.nih.gov/dbvar/studies/nstd186/. Accessed 1 Jul 2021.

12. Collins RL, Brand H, Karczewski KJ, Zhao X, Alföldi J, Francioli LC, et al. A structural variation reference for medical and population genetics. Nature. 2020;581.

13. MacDonald JR, Ziman R, Yuen RKC, Feuk L, Scherer SW. The Database of Genomic Variants: a curated collection of structural variation in the human genome. Nucleic Acids Res. 2014;42 Database issue:D986–92.

14. Navarro Gonzalez J, Zweig AS, Speir ML, Schmelter D, Rosenbloom KR, Raney BJ, et al. The UCSC Genome Browser database: 2021 update. Nucleic Acids Res. 2020;49:D1046–57.

15. Jang YE, Jang I, Kim S, Cho S, Kim D, Kim K, et al. ChimerDB 4.0: an updated and expanded database of fusion genes. Nucleic Acids Res. 2020;48:D817–24.

16. Mitelman F, Johansson B, Mertens F. The impact of translocations and gene fusions on cancer causation. Nature Reviews Cancer. 2007;7:233–45.

17. Tate JG, Bamford S, Jubb HC, Sondka Z, Beare DM, Bindal N, et al. COSMIC: the Catalogue Of Somatic Mutations In Cancer. Nucleic Acids Res. 2019;47:D941–7.

18. Chakravarty D, Gao J, Phillips SM, Kundra R, Zhang H, Wang J, et al. OncoKB: A Precision Oncology Knowledge Base. JCO Precis Oncol. 2017;2017.

19. Gröbner SN, Worst BC, Weischenfeldt J, Buchhalter I, Kleinheinz K, Rudneva VA, et al. The landscape of genomic alterations across childhood cancers. Nature. 2018;555:321–7.

20. Manning G, Whyte DB, Martinez R, Hunter T, Sudarsanam S. The protein kinase complement of the human genome. Science. 2002;298:1912–34.

21. Zhou X, Edmonson MN, Wilkinson MR, Patel A, Wu G, Liu Y, et al. Exploring genomic alteration in pediatric cancer using ProteinPaint. Nat Genet. 2015;48:4–6.

22. McLeod C, Gout AM, Zhou X, Thrasher A, Rahbarinia D, Brady SW, et al. St. Jude Cloud: A Pediatric Cancer Genomic Data-Sharing Ecosystem. Cancer Discov. 2021;11:1082–99.

23. Gao Q, Liang W-W, Foltz SM, Mutharasu G, Jayasinghe RG, Cao S, et al. Driver Fusions and Their Implications in the Development and Treatment of Human Cancers. Cell Rep. 2018;23:227–38.e3.

24. Gough SM, Slape CI, Aplan PD. NUP98 gene fusions and hematopoietic malignancies: common themes and new biologic insights. Blood. 2011;118:6247.

25. Meijerink JPP, Canté-Barrett K, Vroegindeweij E, Pieters R. HOXA-activated early T-cell progenitor acute lymphoblastic leukemia: predictor of poor outcome? Haematologica. 2016;101:654.

26. Matlawska-Wasowska K, Kang H, Devidas M, Wen J, Harvey RC, Nickl CK, et al. MLL Rearrangements Impact Outcome in HOXA-deregulated T-lineage Acute Lymphoblastic Leukemia: A Children's Oncology Group Study. Leukemia. 2016;30:1909.

27. Pacharne S, Dovey OM, Cooper JL, Gu M, Friedrich MJ, Rajan SS, et al. SETBP1 overexpression acts in the place of class-defining mutations to drive FLT3-ITD-mutant AML. Blood Adv. 2021;5:2412–25.

28. Craig AJ, von Felden J, Garcia-Lezana T, Sarcognato S, Villanueva A. Tumour evolution in hepatocellular carcinoma. Nature Reviews Gastroenterology & Hepatology. 2020;17:139–52.

29. Palmer RD, Barbosa-Morais NL, Gooding EL, Muralidhar B, Thornton CM, Pett MR, et al. Pediatric malignant germ cell tumors show characteristic transcriptome profiles. Cancer Res. 2008;68:4239–47.

30. Kubota Y, Seki M, Kawai T, Isobe T, Yoshida M, Sekiguchi M, et al. Comprehensive genetic analysis of pediatric germ cell tumors identifies potential drug targets. Communications Biology. 2020;3:1–11.

31. Tvorogov D, Sundvall M, Kurppa K, Hollmén M, Repo S, Johnson MS, et al. Somatic mutations of ErbB4: selective loss-of-function phenotype affecting signal transduction pathways in cancer. J Biol Chem. 2009;284.

32. Pacenta HL, Allen-Rhoades W, Langenau D, Houghton PJ, Keller C, Heske CM, et al. Prioritization of Novel Agents for Patients with Rhabdomyosarcoma: A Report from the Children's Oncology Group (COG) New Agents for Rhabdomyosarcoma Task Force. J Clin Med Res. 2021;10.

33. Bastidas Torres AN, Melchers RC, Van Grieken L, Out-Luiting JJ, Mei H, Agaser C, et al. Whole-genome profiling of primary cutaneous anaplastic large cell lymphoma. Haematologica. 2021. https://doi.org/10.3324/haematol.2020.263251.

34. Martinez-Monleon A. Molecular and genetic studies in high-risk neuroblastoma. 2021.

35. Barøy T, Kresse SH, Skårn M, Stabell M, Castro R, Lauvrak S, et al. Reexpression of LSAMP inhibits tumor growth in a preclinical osteosarcoma model. Molecular Cancer. 2014;13:93.

36. Saydere AÇ. Analysis of LSAMP gene as a tumor suppressor in neuroblastoma. Bilkent University; 2009.

37. Molenaar JJ, Koster J, Zwijnenburg DA, van Sluis P, Valentijn LJ, van der Ploeg I, et al. Sequencing of neuroblastoma identifies chromothripsis and defects in neuritogenesis genes.

Nature. 2012;483:589–93.

38. Lobbous M, Bernstock JD, Coffee E, Friedman GK, Metrock LK, Chagoya G, et al. An Update on Neurofibromatosis Type 1-Associated Gliomas. Cancers . 2020;12.

39. Hanna JA, Mathkour M, Gouveia EE, Lane J, Boehm L, Keen JR, et al. Pleomorphic Xanthoastrocytoma of the Pineal Region in a Pediatric Patient With Neurofibromatosis Type 1. Ochsner J. 2020;20:226–31.

40. Xie H, Sivaramakrishna Rachakonda P, Heidenreich B, Nagore E, Sucker A, Hemminki K, et al. Mapping of deletion breakpoints at the CDKN2A locus in melanoma: detection of MTAP-ANRIL fusion transcripts. Oncotarget. 2016;7:16490.

41. Kong Y, Hsieh C-H, Alonso LC. ANRIL: A lncRNA at the CDKN2A/B Locus With Roles in Cancer and Metabolic Disease. Front Endocrinol . 2018;9.

42. R2 Genomics Analysis and Visualization Platform. http://hgserver1.amc.nl/cgi-bin/r2/main.cgi. Accessed 12 Jul 2021.
